# Lactylation in Tumor Immune Escape and Immunotherapy: Multifaceted Functions and Therapeutic Strategies

**DOI:** 10.34133/research.0793

**Published:** 2025-09-11

**Authors:** Qing Li, Runkang Zhao, Yang Shen, Dandan Guo, Lvdan Deng, Rongbing Cai, Zhijun Shen, Zhao Xie, Na Hang, Sentao Fu, Dehuan Zhang, Yihang Xu, Zhao Huang, Bufu Tang, Ling Wang

**Affiliations:** ^1^ Department of Oncology, The First Affiliated Hospital of Dalian Medical University, Dalian 116011, PR China.; ^2^ School of Stomatology, Dalian Medical University, Dalian 116044, PR China.; ^3^ Department of Radiation Oncology, Zhongshan Hospital, Fudan University, Shanghai 200032, China.; ^4^ The Second Affiliated Hospital of Dalian Medical University, Dalian 116011, PR China.; ^5^ The First Affiliated Hospital of Dalian Medical University, Dalian 116011, PR China.; ^6^ Hepatic Surgery Center, Tongji Hospital, Tongji Medical College, Huazhong University of Science and Technology, Wuhan, China.; ^7^ Department of Interventional Radiology, Zhongshan Hospital, Shanghai Institute of Medical Imaging, Shanghai Institution of Medical Imaging, Shanghai, National Clinical Research Center of Interventional Medicine, Fudan University, Shanghai 200032, China.

## Abstract

Since its initial identification in 2019, lactylation has emerged as a critical posttranslation modification, attracting substantial research interest due to its diverse roles in biological processes. Lysine lactylation represents a recently characterized posttranslational modification wherein lactate moieties are covalently attached to protein lysine residues through both enzymatic and nonenzymatic pathways. Lactate, a primary glycolytic product, suggests a link between cell metabolism and protein function regulation. In neoplastic tissues, the Warburg effect induces preferential glucose-to-lactate metabolism in cancer cells, establishing hypoxic conditions and elevated lactate concentrations as defining characteristics of the tumor microenvironment. Extensive research has demonstrated lactate’s pivotal role in tumor metastasis and patient outcomes, particularly through its influence on tumor immune microenvironment remodeling, although the precise molecular mechanisms remain under investigation. The characterization of lysine lactylation provides a novel framework for understanding these mechanisms and presents innovative opportunities for therapeutic intervention. This review examines the influence of lactylation on the tumor microenvironment and its effect in various malignancies and explores emerging therapeutic strategies, including genetic manipulation, small-molecule inhibitors, clinical pharmaceuticals, and nanoparticle-based approaches, offering new perspectives in cancer treatment.

## Introduction: Elucidation of Lactylation as a Neoteric Posttranslation Modification in Tumor

Tumor cell development is attributed to the synergistic effects of genetic alterations and metabolic reprogramming. Aberrant metabolic pathways in tumor cells facilitate their enhanced proliferation and metastatic potential. Specifically, altered amino acid catabolism, dysregulated lipid metabolism, and fatty biosynthesis acid oxidation, as well as the maintenance of NADH [reduced form of nicotinamide adenine dinucleotide (oxidized form) (NAD^+^)] metabolism for redox homeostasis, collectively promote accelerated cellular growth [1]. Notably, the up-regulation of glycolysis in tumor cells, even in a phenomenon such as the Warburg effect, is particularly important among these metabolic alterations.

Carl Wilhelm Scheele first isolated lactic acid from sour milk in 1780. Under physiological pH, lactic acid predominantly exists in its deprotonated form, known as lactate. The period spanning from the early observations of lactic acid by von Muralt in the late 19th century to the seminal muscle experiments conducted by Fletcher and Hopkins in 1907 is commonly referred to as the “pre-lactic acid era”. During this era, researchers established a positive correlation between lactate accumulation and the intensity of glycolysis in muscle tissue [2]. The lactate shuttle has elucidated the role of lactate in facilitating the transfer of glycolytic substrates and mediating cell signal transduction [3], thereby establishing a link between glycolytic and oxidative pathways. Brooks further demonstrated the utilization of lactate under aerobic conditions, challenging long-held misconceptions about lactate metabolism [4]. In the 1920s, Otto Warburg observed that tumor cells preferentially produced large amounts of lactate through glycolysis under normoxic conditions, a phenomenon later termed the “Warburg effect” [5,6]. However, the precise mechanisms by which the Warburg effect contributes to cancer progression remain an active area of investigation.

**Fig. 1.**
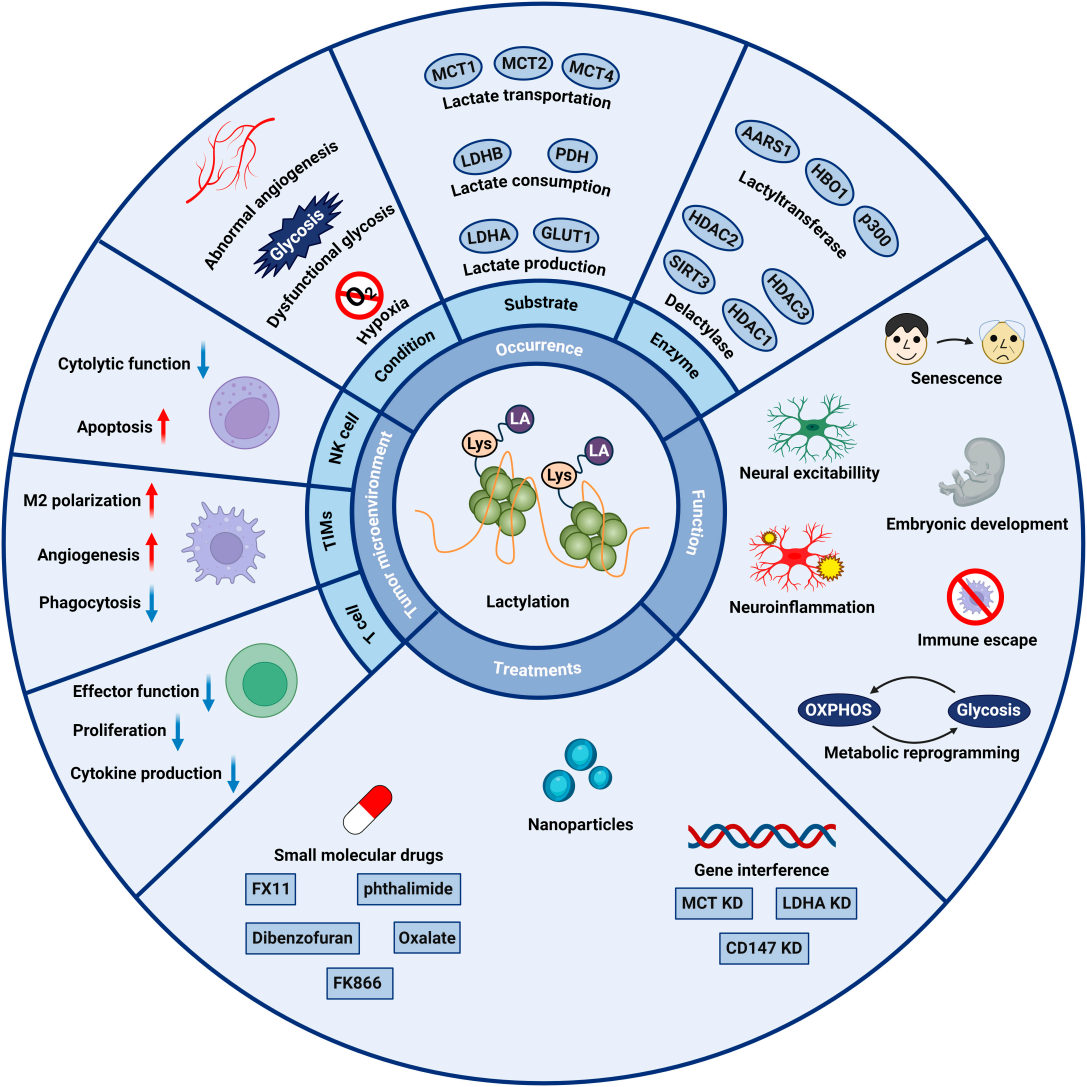
Comprehensive overview of lactylation in tumor pathogenesis. This schematic delineates the mechanistic basis of lactylation modifications, their physiological functions in nonneoplastic contexts, potential therapeutic targets, and their multifaceted effects on the TME. In neoplastic tissues, lactylation modifications are predominantly driven by augmented glycolytic activity and the hypoxic milieu characteristic of the TME. Lactylation modifications exert diverse and cell type-specific effects on the functional capabilities and developmental trajectories of various immune cell populations within the TME. Emerging evidence suggests that modulation of lactylation processes may represent a promising avenue for novel therapeutic interventions in oncology.

A seminal study published in 2019 demonstrated that lactate-mediated lactylation of specific lysine residues functions as a histone modification that directly regulates gene transcription at the chromatin level. This posttranslational modification, analogous to acetylation, methylation, phosphorylation, and other histone marks, has the potential to modulate the expression of various oncogenes and tumor suppressor genes [7]. Accordingly, this review aims to elucidate the role of lactylation within the tumor immune microenvironment and discuss its potential implications for cancer immunotherapy (Fig. 1).

## Lactate Metabolism and Lactylation: Mechanisms and Implications in Tumor

### Lactate homeostasis in tumor: Production, utilization, and clearance mechanisms

The primary pathway for lactate production is through glucose glycolysis, as a byproduct of glucose metabolism. When in an oxygen-deficient environment, the pyruvate produced from glucose through a series of pathways instead of entering the mitochondrial tricarboxylic acid (TCA) cycle, it is converted into lactate acid through lactate dehydrogenase (LDH) [8]. If lactate accumulates, symptoms of lactic acidosis may occur, necessitating the removal of excess lactate. When oxygen conditions improve or intense exercise ceases, lactate is converted into pyruvate under the action of lactate dehydrogenase, then enters the TCA cycle via pyruvate dehydrogenase to participate in aerobic reactions, or undergoes gluconeogenesis to convert into glucose [9].

In cancer cells, another pathway for lactate production is the breakdown metabolism of glutamine [10]. Warburg observed that tumor cells in aerobic conditions still secrete glucose in the form of lactate [5]. Using ^13^C nuclear magnetic resonance (NMR) spectroscopy, researchers have examined the metabolic activity of glioblastoma cells. These studies revealed the coexistence of aerobic glycolysis and an active TCA cycle. This led to the discovery of elevated glutamine metabolism in these cells, in which carbon derived from glutamine exits the mitochondria, is converted to pyruvate in the cytoplasm, and subsequently contributes to lactate production [10].

### Stereochemistry and biological significance of lactate isomers in tumor metabolism

Due to the stereochemistry of its chiral carbon atom, lactate has 3 forms of isomers in organisms: racemic dl-lactate, d-lactate, and l-lactate.

l-Lactate is the predominant stereoisomer in the human body. Under anaerobic conditions, glucose is primarily metabolized to pyruvate in skeletal muscle fibers and subsequently reduced to lactate through a NADH-dependent pathway, generating adenosine triphosphate (ATP). The accumulation of lactate can lead to lactic acidosis. LDH catalyzes the interconversion between lactate and pyruvate, thereby regulating this metabolic process. LDH catalyzes both the forward and reverse reactions in this reversible redox process [11]. In hepatocellular carcinoma (HCC) cells, lactate accumulation creates an acidic microenvironment, compromising the activity of l-lactate dehydrogenase [12].

d-Lactate is predominantly found in lower organisms, originating from 2 primary sources: the metabolism of carbohydrates and lipids [13], and production by intestinal microbiota [14]. d-Lactate facilitates the transport of certain metabolic substrates. Research has identified 3 transport proteins that transfer d-lactate to the inner mitochondrial membrane. The metabolism and excretion pathways of d-lactate remain subjects of ongoing debate in the scientific community [15]. Elevated d-lactate levels have been observed in both urine and blood samples of diabetic patients [16]. Conversely, reduced d-lactate metabolism in Alzheimer’s disease (AD) patients may serve as a potential biomarker [17].

### Multifaceted functions of lactate in tumor: From energy metabolism to signaling molecule

#### Providing and balancing energy

Lactate was traditionally considered a mere byproduct of anaerobic glycolysis, often regarded as metabolic waste. However, recent studies have revealed that lactate, along with pyruvate, plays a pivotal role in maintaining intracellular redox homeostasis by buffering the NAD^+^/NADH ratio [18]. Accumulating evidence confirms that glucose is the primary energy substrate for cerebral metabolism. In nontumor tissues such as the brain, lactate can also act as an alternative energy substrate under glucose deprivation and modulate neuronal excitability, particularly in proopiomelanocortin (POMC) neurons [19,20]. Furthermore, lactate promotes both the release and mitochondrial uptake of magnesium ions, thereby supporting mitochondrial bioenergetics [21].

Importantly, within the tumor microenvironment (TME), lactate functions not only as a metabolic byproduct but also as a versatile energy mediator, facilitating metabolic cooperation among heterogeneous tumor and stromal cell populations. Tumors are intrinsically characterized by both spatial and metabolic heterogeneity [22]. A hallmark of rapidly proliferating solid tumors is their tendency to outpace neovascularization, leading to localized hypoxia, especially in cells situated far from functional vasculature [23]. These hypoxic tumor cells primarily depend on anaerobic glycolysis, converting pyruvate to lactate via lactate dehydrogenase A (LDHA), followed by export through monocarboxylate transporter 4 (MCT4). In contrast, tumor cells in well-oxygenated regions, typically adjacent to vasculature, utilize lactate as an oxidative substrate. These cells express elevated levels of MCT1 and lactate dehydrogenase B (LDHB), enabling efficient lactate import and subsequent conversion to pyruvate for entry into the TCA cycle [24,25]. This spatially organized metabolic compartmentalization establishes an intratumoral lactate shuttle, enhancing metabolic flexibility and supporting tumor persistence under nutrient-deprived conditions. Beyond tumor cells, cancer-associated fibroblasts (CAFs)—the predominant nonmalignant stromal component in the TME—also actively engage in this metabolic crosstalk [26]. Under stimulation by diverse cytokines and extracellular matrix (ECM) remodeling signals, normal fibroblasts undergo metabolic reprogramming, giving rise to the CAF phenotype. Compared to their quiescent counterparts, CAFs show up-regulated glucose uptake, heightened glycolytic flux, and enhanced lactate production [27]. These metabolic adaptations allow CAFs to deliver lactate or ketone bodies to neighboring tumor cells, thereby sustaining tumor proliferation and survival through a mechanism known as the “reverse Warburg effect” [26]. This lactate-driven metabolic symbiosis buffers intracellular acidity and serves as a flexible energy reservoir, thereby facilitating tumor progression under hypoxic or nutrient-limited conditions.

#### Fatty acid metabolism

Metabolic reprogramming is a defining hallmark not only of cancer cells but also of the surrounding stromal and immune cell populations [28]. One emerging and increasingly appreciated aspect of this reprogramming is the metabolic crosstalk between lactate metabolism and lipid biosynthesis. Lactate, produced in large quantities through aerobic glycolysis (i.e., the Warburg effect), serves as a major carbon source for generating acetyl-coenzyme A (CoA)—a central precursor in de novo fatty acid biosynthesis. This metabolic shift is particularly evident under hypoxic or glucose-restricted conditions, where lactate becomes a preferred anabolic substrate for lipid synthesis in both tumor and stromal cells [29–31]. Consequently, elevated lactate levels have been shown to enhance fatty acid biosynthesis [32].

Mechanistically, lactate-derived acetyl-CoA facilitates fatty acid biosynthesis by activating ATP citrate lyase (ACLY) and acetyl-CoA carboxylase (ACC), thereby promoting membrane formation, energy storage, and the generation of signaling lipids essential for rapid tumor proliferation [33,34]. Additionally, lactate can activate sterol regulatory element-binding protein 2 (SREBP2) in tumor-infiltrating dendritic cells (DCs)—a master transcriptional regulator of lipogenesis—thereby further amplifying lipid metabolic pathways [35]. In inflammatory microenvironments, lactate accumulation up-regulates the expression of the lactate transporter SLC5A12 on CD4^+^ T cells. This phenotypic shift in CD4^+^ T cells establishes a positive feedback loop that promotes fatty acid synthesis [36]. The convergence of lactate metabolism and fatty acid biosynthesis constitutes a critical metabolic–immune interface within the TME. This metabolic axis not only sustains tumor cell survival under metabolic stress but also reprograms immune cells into a tumor-supportive phenotype, thereby highlighting promising targets for metabolic intervention in cancer therapy.

#### Role of lactate as a signal molecule

Even before the discovery of histone lactylation, numerous studies had already established that lactate exerts diverse nonmetabolic functions, including the regulation of inflammation, tumor progression, and oxidative stress [3]. Among the approximately 800 members of the G protein-coupled receptor (GPCR) family, GPR81—also known as hydroxycarboxylic acid receptor 1 (HCAR1)—has emerged as a highly selective receptor that senses extracellular lactate. It is widely expressed in various cell types, such as neurons, adipocytes, cancer cells, and retinal cells [37–40]. Within the TME, which is frequently characterized by elevated glycolytic activity, excessive lactate accumulates in the extracellular space and activates GPR81 on the surface of tumor cells [41]. In multiple tumor models, pharmacological or genetic inhibition of GPR81 has been shown to significantly suppress cancer cell proliferation and metastasis. For example, in lung cancer, the blockade of either lactate production or GPR81 signaling leads to down-regulation of PD-L1 expression, thereby enhancing treatment sensitivity and reducing drug resistance [42]. Similarly, in both pancreatic and HCCs, silencing GPR81 markedly impairs cellular proliferation and metastatic capacity [43]. In colorectal cancer (CRC), lactate activates the GPR81/adenosine 3′,5′-monophosphate (cAMP)/protein kinase A (PKA)/CREB (cAMP response element-binding protein) signaling axis, which suppresses MLH1 expression, thereby impairing mismatch repair processes and promoting chemoresistance [44]. Likewise, in cervical cancer, GPR81 knockdown reduces the expression of ABCB1 and increases sensitivity to doxorubicin [45]. In breast cancer, GPR81 inhibition restores major histocompatibility complex (MHC) class II expression in DCs, effectively reversing immune suppression and enhancing antitumor immunity [42]. Furthermore, inhibition of GPR81 signaling via the phosphatidylinositol 3-kinase (PI3K)/Akt/cAMP pathway has been reported to down-regulate amphiregulin (AREG), a key pro-angiogenic factor [46]. Given that PI3K/Akt activation is known to transcriptionally up-regulate vascular endothelial growth factor (VEGF) expression, it is plausible that GPR81-mediated lactate signaling contributes to tumor angiogenesis through a VEGF-dependent mechanism [46,47].

GPR81 signaling is also implicated in epithelial–mesenchymal transition (EMT), a critical process in tumor metastasis. In esophageal cancer cells, lactate-induced activation of the GPR81/Wnt/β-catenin pathway promotes EMT [48]. In CRC, GPR81-mediated degradation of DEPDC5 facilitates EMT and is closely associated with metastatic progression [49]. Additionally, GPR81 knockdown has been found to impair the transforming growth factor-β (TGF-β)/Smad signaling axis in models of pulmonary fibrosis [50]. Given the well-established role of TGF-β as a pleiotropic cytokine that promotes both EMT and early tumor progression, it is reasonable to hypothesize that GPR81-mediated lactate signaling may influence EMT through TGF-β–related mechanisms [51]. Beyond its role in tumor cells, GPR81 also functions as a metabolic sensor and immunomodulatory molecule. Evidence suggests that activation of signal transducer and activator of transcription 3 (STAT3) contributes to the immunosuppressive effects mediated by GPR81 signaling [52]. Interestingly, interleukin-6 (IL-6)/STAT3 signaling has also been implicated in EMT within tumor-infiltrating myeloid cells (TIMs), CAFs, and tumor-associated adipocytes [53–55], suggesting that STAT3/IL-6 pathways may jointly participate in GPR81-mediated EMT. Recent findings have expanded the functional relevance of GPR81 beyond cancer biology. Specifically, GPR81 expression in adipose tissue has been linked to tumor-induced cachexia, where it contributes to both adipose and muscle wasting, ultimately worsening patient prognosis and reducing survival [56].

Taken together, these findings support the notion that lactate acts as a signaling molecule by serving as a ligand for GPR81, orchestrating a wide spectrum of downstream effects including immune evasion, metabolic reprogramming, chemoresistance, angiogenesis, and EMT. The subsequent identification of lactylation has further bridged lactate metabolism with epigenetic regulation. In the following section, we will explore the role of lactylation in tumor biology and discuss how targeting lactate-derived modifications may offer translational insights for future cancer therapies.

### Molecular mechanisms and regulation of protein lactylation in tumor

**Fig. 2.**
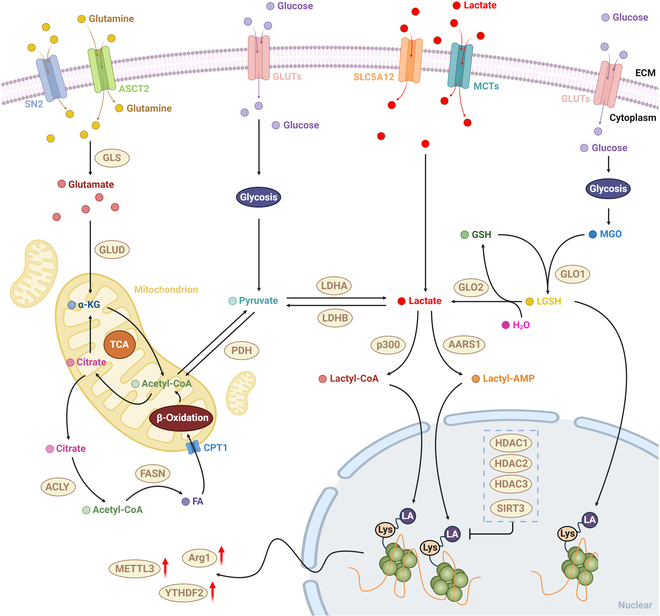
Mechanistic pathways of enzymatic and nonenzymatic lactylation processes in tumor. In neoplastic cells, the precursor for lactylation modifications, lactate, is primarily derived from 3 metabolic pathways: glutaminolysis, glycolysis, and extracellular lactate uptake. Glutamine is internalized via the SN2 and ASCT2 transporters, subsequently undergoing deamidation glutamated catalyzed through glutaminase (GLS). Glutamate is further metabolized to α-ketoglutarate (α-KG) by glutamate dehydrogenase (GLUD); after that, it enters the mitochondrial TCA cycle. α-KG through the TCA cycle can be metabolized to acetyl-CoA, while cytosolic citrate can undergo fatty acid synthesis. These fatty acids can then be changed into mitochondria via carnitine palmitoyltransferase I (CPT1) for β-oxidation, yielding acetyl-CoA. This acetyl-CoA is transformed to lactate through the sequential actions of pyruvate dehydrogenase (PDH) and lactate dehydrogenase A (LDHA). Glucose, conversely, is internalized via various glucose transporter (GLUT) isoforms and undergoes glycolysis, culminating in lactate production. Extracellular lactate can be directly imported as a precursor for lactylation modifications via the sodium/glucose cotransporter SLC5A12 and members of the MCT family. Lactate can undergo lactylation modifications through both enzymatic and nonenzymatic mechanisms. Two enzymes have been identified to catalyze lactylation modifications: p300, which utilizes acetyl-CoA as a donor, and alanyl-tRNA synthetase (AARS1), which synthesizes lactoyl-AMP from ATP and lactate. Both enzymes facilitate histone lactylation. In nonenzymatic pathways, the glycolytic byproduct MGO rapidly conjugates with GSH to form lactoylglutathione (LGSH), a reaction catalyzed by GLO1. LGSH can subsequently act as an acyl donor, nonenzymatically transferring lactoyl groups to lysine residues on target proteins, resulting in protein lactylation. Histone lactylation modifications can modulate the transcription of various genes, including ARG1, YTHDF2, and METTL3, thereby eliciting specific biological responses.

Protein lactylation occurs via 2 mechanistically distinct pathways: an enzymatic pathway that is tightly regulated, and a nonenzymatic pathway driven by metabolic byproducts. In the enzymatic pathway, the histone acetyltransferase p300 catalyzes the transfer of lactyl groups from lactyl-CoA—derived from l-lactate—to specific lysine residues, particularly on histones [7]. This modification is reversible and dynamically regulated, with histone deacetylases HDAC1 to HDAC3 functioning as delactylases; among them, HDAC3 exhibits a stronger activity toward l-lactylated sites than d-lactylated ones [57]. Enzymatic lactylation is generally site-specific and plays a key role in transcriptional reprogramming and epigenetic regulation in response to metabolic signals. In contrast, nonenzymatic lactylation arises spontaneously, without enzymatic catalysis, through a metabolite-driven acyl transfer process. A well-characterized route involves the glycolytic byproduct methylglyoxal (MGO), which reacts with glutathione (GSH) via glyoxalase I (GLO1) to form *S*-d-lactoylglutathione (SLG). SLG is normally hydrolyzed by glyoxalase II (GLO2) to regenerate GSH and produce d-lactate. However, under conditions of GLO2 down-regulation—such as nuclear factor κB (NF-κB)-mediated inflammation or metabolic stress—SLG accumulates in the cytosol. This accumulation enables SLG to act as a reactive acyl donor, transferring its d-lactoyl group to lysine residues on nearby proteins via a nonenzymatic mechanism, often facilitated by adjacent cysteine residues [58] (Fig. 2).

The biological implications of these 2 pathways differ significantly. Enzymatic lactylation is tightly regulated and contributes to precise transcriptional control and chromatin remodeling. In contrast, nonenzymatic lactylation may act as a broader metabolic sensor that passively links glycolytic flux to protein structure and function, often attenuating inflammatory signaling or metabolic enzyme activity. Together, these dual modes of lactylation highlight the multifaceted role of lactate—not only as a metabolic end-product but also as a key modulator of protein function and tumor cell behavior within the TME.

## Lactylation-Induced Phenotypic and Functional Alterations in Tumor: Implications for Tumor Progression

Lactate functions as a signaling molecule in the GPCR pathway. It has 2 mechanisms: autocrine and paracrine. Lactate secreted by cancer cells activates GPR81 on the same cancer cells in the autocrine pathway. The paracrine pathway, conversely, involves lactate from cancer cells activating GPR81 on diverse cell types in the TME, such as immune cells, endothelial cells, and adipocytes. Activation of GPR81 consequently promotes multiple tumorigenic processes, including angiogenesis, immune evasion, and chemoresistance [38].

### Relationship with tumor formation and invasion

Tp73 is a kind of homolog in the tumor suppressor p53 that is frequently overexpressed in tumors, conferring a propagating advantage to cancer cells. This protein augments glycolysis, thereby potentiating the Warburg effect [59,60].

GPR81, a lactate receptor primarily found in adipose and muscle tissues, is activated by lactate and highly up-regulated in most cancer cells [42,43,61]. This up-regulation facilitates tumor cell hyperplasia and survival. For instance, in lactate-rich lung cancer microenvironments, lactate stimulates the rise of PD-L1 (programmed death-ligand 1) expression, a process mediated by GPR81. Conversely, GPR81 signaling silencing in cancer cells causes reduced PD-L1 protein levels and suppression of PD-L1 promoter activity [62]. Consequently, lactate secretion by tumor cells modulates GPR81 signaling, significantly impacting tumor growth and immune evasion.

Tumor-derived lactate generates an acidic extracellular environment through the action of carbonic anhydrase-IX (CA-IX) and sodium-hydrogen exchanger-1 (NHE1) [63]. This acidic milieu significantly impacts tumor cell survival and proliferation. In pancreatic cancer cells, for example, LAMC2 (laminin subunit gamma-2) stimulates Akt-Ser^473^ phosphorylation, up-regulating NHE1 expression. This cascade triggers the formation of actin-dependent dynamic pseudopods and initiates the EMT program, enhancing tumor cell invasiveness [64]. Analogous mechanisms have been documented in various cancer types, including bladder, breast, and gastric cancers [65–70].

**Fig. 3.**
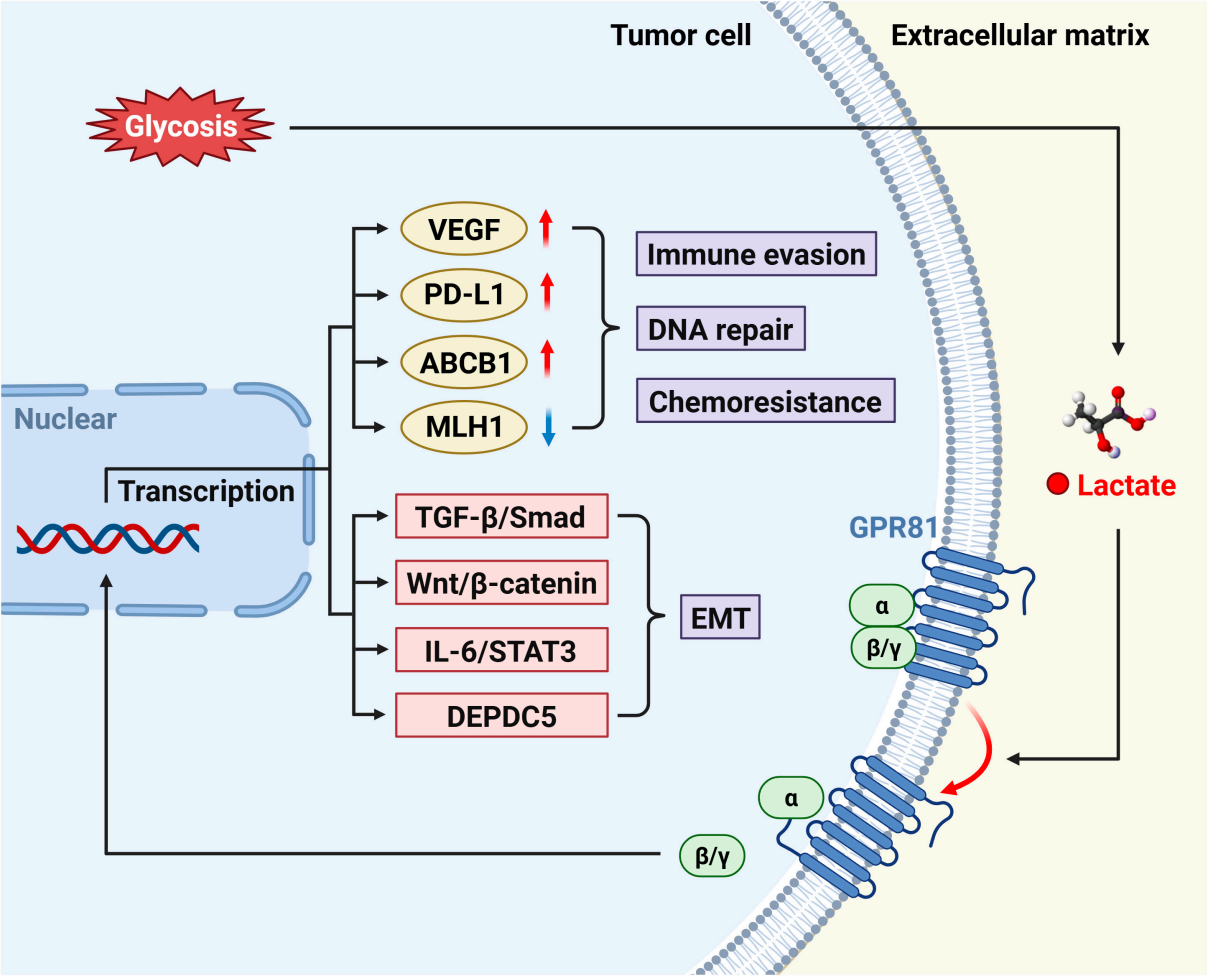
Multifaceted roles of lactate in tumorigenesis and progression. Canonically, augmented glycolytic activity in neoplastic tissues promotes excessive lactate production within the TME, fostering the development of a hypoxic and acidic milieu. Lactate, via activation of its cognate receptor GPR81, induces the expression of multiple genes and initiates downstream signaling cascades, culminating in diverse oncogenic processes including immune evasion, enhanced DNA repair mechanisms, chemoresistance, and EMT.

Lactate is a kind of pleiotropic signaling molecule, orchestrating protein turnover and modulating many signaling cascades, for example, TGF-β/Smad, Wnt/β-catenin, IL-6/STAT3, and hepatocyte growth factor/mesenchymal–epithelial transition factor (HGF/MET) pathways. These interconnected signaling networks orchestrate critical processes in tumor progression, such as basement membrane rebuilding and EMT, ultimately promoting invasive phenotypes [71] (Fig. 3).

### The relationship between lactylation and tumor immune evasion and suppression

Tumor immune evasion encompasses mechanisms by which neoplastic cells circumvent immune surveillance, subvert immune processes, and foster a microenvironment permissive to tumor growth [72].

Lactate efflux from tumor cells generates an acidic TME [63]. Elevated lactate concentrations suppress the nuclear factor of activated T cells (NFAT) in T and natural killer (NK) cells, attenuating interferon-γ (IFN-γ) production and facilitating immune evasion [73]. Concurrently, lactate profoundly impairs T cell propagation and cytokine production, the proportion up to 95%, compromising their cytotoxic and lytic activities [74]. Furthermore, lactate induces macrophage polarization from the pro-inflammatory M1 type to the immunosuppressive M2 type, thereby reducing immune responses within the TME [7,75].

Monocytic myeloid-derived suppressor cells (M-MDSCs) play a crucial role in tumor immune evasion. Lactate up-regulates METTL3 (methyltransferase-like 3) expression in TIMs via H3K18 lactylation. METTL3 mediates m6A modification of Jak1 (Janus kinase 1) mRNA in TIMs. This augmentation of the m6A–YTHDF1 axis modulates Jak1 protein translation and STAT3 phosphorylation. Moreover, 2 lactylation sites have been found in the zinc finger domain of METTL3 [76]. Consequently, lactylation critically enhances the immunosuppressive capacity of TIMs.

In prostate cancer, PTEN (phosphatase and tensin homolog) loss leads to up-regulation of the PI3K signaling pathway. PI3K facilitates lactate production and tumor-associated macrophage (TAM) immunosuppression [77].

As discussed above, this section specifically focuses on lactate-based tumor escape mechanisms rather than lactylation-mediated strategies. Although lactate can induce lactylation, it is important to note that lactate levels do not always correlate with the extent of protein lactylation. Given the intricate relationship between tumor escape mechanisms and the tumor immune microenvironment (TIME), the direct effects of protein lactylation on tumor immune evasion will be comprehensively analyzed and summarized in subsequent chapters.

## Targeting Lactylation to Modulate Immune Responses in Tumor: Therapeutic Implications and Strategies

### The impact of lactylation on various types of tumors

#### Hepatocellular carcinoma

HCC is the predominant form of primary liver cancer and a major contributor to global cancer-related mortality [78]. While early-stage HCC is often amenable to surgical resection, advanced disease typically requires a multimodal approach involving chemotherapy, targeted therapy, and immunotherapy [79]. HCC is known for its remarkable metabolic adaptability. Compared with normal hepatocytes that depend primarily on oxidative phosphorylation, HCC cells rewire their metabolism to favor aerobic glycolysis, even under normoxic conditions. In parallel, they exhibit increased glutamine anaplerosis, de novo fatty acid synthesis, and enhanced serine–glycine one-carbon metabolism [80–82]. This metabolic reprogramming supports rapid tumor growth and biosynthesis while also promoting immune evasion and resistance to therapy [83].

One consequence of this glycolytic shift is the excessive production and accumulation of lactate. Beyond serving as a metabolic byproduct, lactate has emerged as a key regulator of gene expression through posttranslational modifications such as lactylation. Recent studies suggest that lactylation contributes to HCC progression by linking metabolic activity to epigenetic and functional changes in tumor cells. Sirtuin 3 (SIRT3), a mitochondrial delactylase, is expressed in HCC and acts as a tumor suppressor by promoting apoptosis and inhibiting tumor growth. Proteomic analyses have identified widespread lactylation in virus-associated HCC, particularly on metabolic enzymes involved in glycolysis and the TCA cycle [84]. These findings indicate that lactylation may function as a metabolic rheostat, sustaining anabolic metabolism and reinforcing the Warburg effect. In addition to modifying metabolic enzymes, lactylation also influences the epigenetic landscape of HCC. A recent study identified demethylzeylasteral as a novel anticancer agent that suppresses liver cancer stem cell formation by targeting histone lactylation at H3K9la and H3K56la [85]. As liver cancer stem cells are closely linked to tumor recurrence and treatment resistance, targeting lactylation at specific histone sites may offer a promising strategy to reduce tumor-initiating potential [86].

#### Colorectal cancer

CRC arises in a unique metabolic and immunological context shaped by the gut microbiota [87]. Short-chain fatty acids (SCFAs), such as butyrate, play dual roles in epithelial homeostasis and tumor progression. Notably, butyrate has been shown to suppress METTL3 expression, thereby influencing RNA methylation patterns and modulating immune evasion in CRC. This epigenetic regulation affects the TIM population, which plays a key role in shaping an immunosuppressive microenvironment [88,89]. Beyond SCFAs, recent findings highlight the role of tumor-resident microbiota in shaping CRC progression. Specifically, intratumoral *Escherichia coli* has been shown to enhance glycolysis in cancer cells, leading to increased lactate production. This lactate, in turn, induces RIG-I lactylation in macrophages, suppressing NF-κB activation and NLRP3 inflammasome signaling. The resulting M2 macrophage polarization promotes an immunosuppressive microenvironment and facilitates liver metastasis [90].

Under hypoxic conditions—a hallmark of the tumor core—CRC cells exhibit elevated glycolytic activity, leading to excess lactate accumulation [91]. Lactate has been shown to enhance the stability of β-catenin, a protein frequently overexpressed in CRC [92]. Stabilized β-catenin subsequently activates the Wnt/β-catenin signaling pathway, thereby promoting tumor cell proliferation, invasion, and metastasis [93]. Although this effect was initially attributed solely to signaling modulation, emerging evidence suggests that lactate may also exert its effects through posttranslational modification (PTM) mechanisms, particularly lactylation.

#### Non-small cell lung cancer

Non-small cell lung cancer (NSCLC), accounting for over 80% of all lung cancer cases, is characterized by profound metabolic reprogramming that supports tumor growth and therapeutic resistance [94]. Emerging studies have revealed that lactate plays a dual regulatory role in NSCLC metabolism and gene expression.

On the one hand, exogenous lactate reduces glycolytic flux by down-regulating HK1 and PKM while enhancing mitochondrial metabolism through up-regulation of SDHA and IDH3G. These changes are partly mediated by increased histone lactylation at gene promoters, linking lactate to transcriptional control of metabolic enzymes [95].

On the other hand, in NSCLC cells resistant to epidermal growth factor receptor tyrosine kinase inhibitors (EGFR-TKIs), lactate contributes to a positive feedback loop with NNMT and ALDH3A1. NNMT reduces histone methylation at EGR1 and ALDH3A1 promoters, promoting their expression. ALDH3A1 enhances lactate production via LDHA, which in turn increases H3K18 lactylation on the NNMT promoter through p300, sustaining NNMT up-regulation. This NNMT/ALDH3A1/lactate axis forms a self-reinforcing loop that promotes EGFR-TKI resistance and tumor proliferation [96]. Notably, global histone lactylation and H3K18la levels are significantly elevated in tumor tissues and cells from TKI-resistant NSCLC patients compared with TKI-sensitive counterparts. These modifications predominantly localize to the nucleus and are positively correlated with poor prognosis. Pharmacological inhibition of NNMT in combination with osimertinib has demonstrated synergistic antitumor effects in resistant xenograft models, highlighting the therapeutic potential of targeting lactate-driven epigenetic regulation [96].

Taken together, lactate in NSCLC not only reprograms cellular metabolism but also serves as a key epigenetic modulator that contributes to therapeutic resistance, especially through histone lactylation-mediated transcriptional regulation.

#### Melanoma

Melanoma, particularly its cutaneous and uveal subtypes, demonstrates significant metabolic plasticity, characterized by high glycolytic activity and lactate accumulation. Unlike many other solid tumors, melanoma cells often retain functional mitochondria, enabling them to flexibly switch between oxidative phosphorylation and aerobic glycolysis depending on nutrient and oxygen availability. This metabolic adaptability supports rapid growth, immune escape, and metastatic dissemination [97–99].

Recent studies have highlighted lactate not only as a metabolic byproduct but also as an epigenetic regulator in melanoma. In ocular melanoma, elevated lactate levels correlate with poor clinical outcomes and may serve as actionable biomarkers [100]. Mechanistically, lactate-induced histone lactylation enhances the transcription of YTHDF2, an m6A reader protein that recognizes and destabilizes methylated transcripts of PER1 and TP53—2 genes involved in circadian rhythm and tumor suppression, respectively. This degradation impairs cellular homeostasis, dampens apoptotic responses, and promotes tumorigenesis [101]. These findings suggest that lactate-driven lactylation in melanoma reprograms gene expression at both the epigenetic and posttranscriptional levels, contributing to tumor initiation and progression. Targeting this lactate–lactylation–YTHDF2 axis may offer novel therapeutic avenues for metabolically active melanomas.

#### Renal cancer

Renal cell carcinoma (RCC) is the most common malignancy of the urinary tract, with clear cell RCC (ccRCC) accounting for approximately 75% of cases and exhibiting particularly poor prognosis [102,103]. A hallmark of ccRCC is loss-of-function mutations in the von Hippel–Lindau (VHL) gene, which drive metabolic reprogramming toward aerobic glycolysis and excessive lactate production [104,105]. Recent studies have shown that lactate accumulation in ccRCC not only reflects altered metabolism but also contributes to tumor progression through epigenetic regulation. Specifically, VHL inactivation enhances histone H3K18 lactylation at the platelet-derived growth factor receptor β (PDGFRβ) promoter, up-regulating its expression and promoting cell proliferation and migration. This lactate–H3K18la–PDGFRβ axis establishes a positive feedback loop that sustains oncogenic signaling [106]. Consequently, therapeutic strategies targeting the disruption or modulation of this feedback loop present promising avenues for ccRCC treatment.

#### Comparative analysis and clinical perspectives

**Fig. 4.**
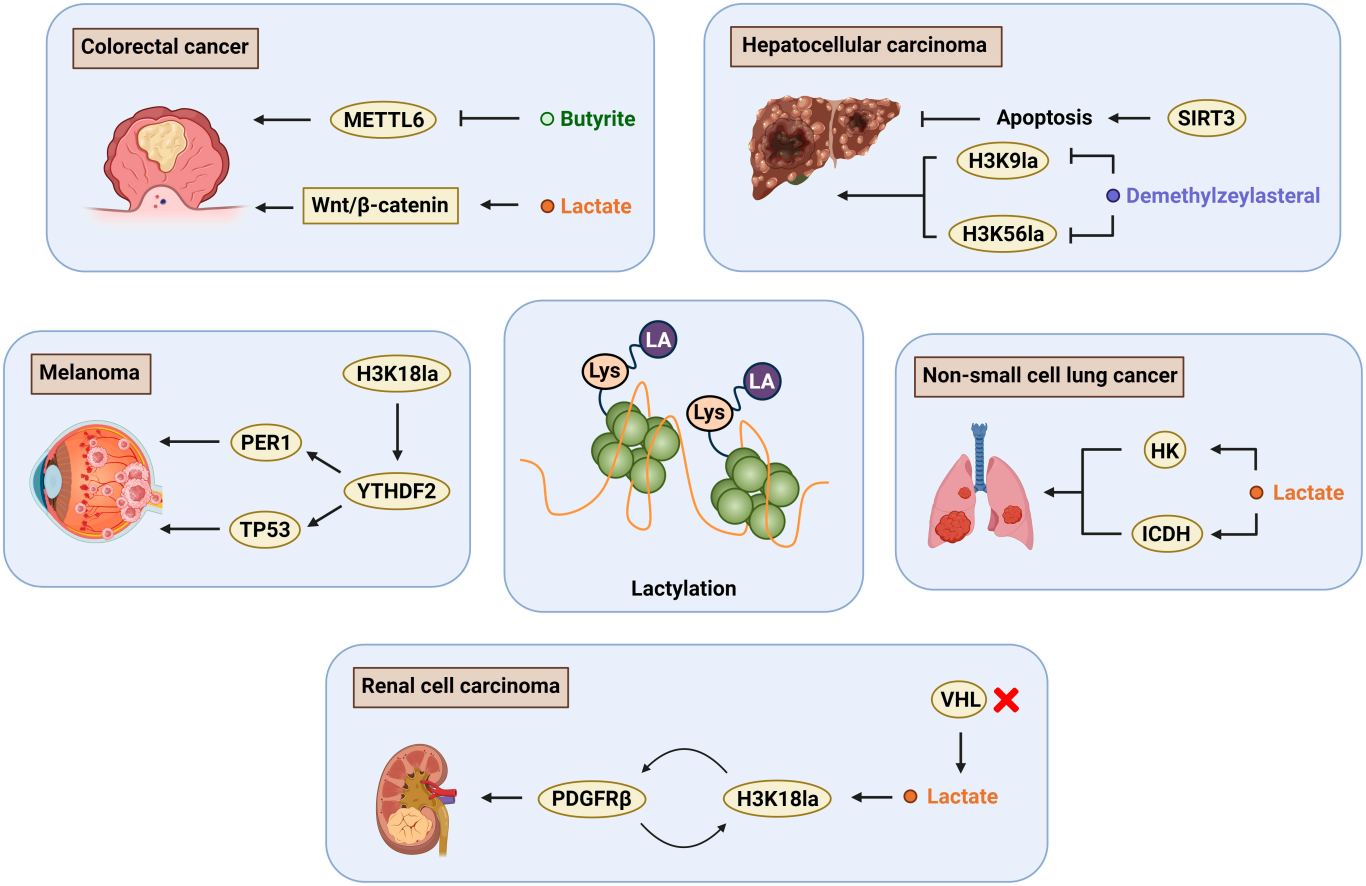
Differential impact of lactylation across diverse cancer types with emphasis on tumor. Histone lactylation modifications have been implicated as pivotal epigenetic regulators in diverse malignancies, including melanoma, colorectal adenocarcinoma, HCC, renal cell carcinoma, and non-small cell lung carcinoma. Furthermore, certain small-molecule therapeutics demonstrate antineoplastic efficacy through metabolic modulation or direct targeting of lactylation sites, thus presenting novel avenues for intervention in cancer treatment.

Histone lactylation has emerged as a shared regulatory mechanism across multiple tumor types. In all 5 cancers reviewed, lactate acts not only as a metabolic byproduct but also as an epigenetic modulator that shapes gene expression and tumor behavior. A common feature is the link between elevated glycolysis and increased lactate levels, which drive histone lactylation at key promoter regions. This modification activates genes involved in metabolism (HCC, ccRCC), immune evasion (CRC), therapy resistance (NSCLC), and transcript stability (melanoma). However, tumor-specific patterns are evident. In HCC, lactylation supports stemness and metabolic enzyme activation. CRC shows lactylation–microbiota interactions influencing immune escape. NSCLC displays a self-reinforcing loop involving NNMT, ALDH3A1, and H3K18la, contributing to drug resistance. In melanoma, lactylation up-regulates YTHDF2, promoting mRNA decay of tumor suppressors. ccRCC links VHL loss to lactate-driven PDGFRβ activation through H3K18la (Fig. 4). Despite different targets, positive feedback loops involving lactate and lactylation are recurrent. These loops sustain oncogenic signaling and contribute to poor prognosis.

Clinically, lactylation-related markers such as H3K18la and YTHDF2 may serve as prognostic indicators or therapeutic targets. Inhibitors of lactylation regulators (e.g., p300 and NNMT) show promise in preclinical models. Future therapies may benefit from combining metabolic blockades with epigenetic modulation.

Overall, lactylation bridges metabolism and chromatin regulation. Its context-specific roles offer both mechanistic insight and potential for targeted interventions in cancer treatment (Table 1).

**Table 1.** Comparative analysis of lactylation-mediated molecular mechanisms in tumor: Implications for pan-cancer therapeutic strategies

Cancer types	Lactylation modification site	Deacetylase	Acyltransferase	Protein modification effect/target gene	Impact on tumors	Cite
Hepatocellular carcinoma	CCNE2 K348la	SIRT3	/	Regulation of the cell cycle and cell growth	Dissemination, migration, and invasion, inhibiting the therapeutic effects of berberine on tumors	[205]
	AK2 K28la	HDAC	p300	Involvement in energy disruption and malignant cell transformation	Facilitated the development of HCC cells, increased tumor thrombosis susceptibility, and related to poor prognosis in HCC patients	[84]
	H3K9la, H3K56la	/	/	Promotion of cell cycle-related protein expression, leading to abnormal glycolysis/gluconeogenesis pathways, and maintaining the happening of liver cancer stem cells	Stimulated cell progress and colony form, enhanced cell migration ability, and involved in decitabine’s treatment of liver cancer	[85]
	ALDOA K230/322la	/	P300	Facilitated DDX17 nuclear translocation to promote the stemness of liver cancer hepatocytes and enhance cell glycolytic capacity	Directly regulated the proliferation, migration, and stem cell characteristics of LCSCs and other malignant biological behaviors	[221]
	H3K56la	/		Facilitated the expression of SOX2 and OCR4 to maintain the stemness of cancer cells		
Colorectal cancer	H4K8la	/	/	Inhibited the binding of transcription factor YY1 with the promoter to induce the erise of the noncoding long chain RNA LINC00152	Involved in the impact of Gram-positive bacteria on the development of colorectal cancer cells	[222]
	β-Catenin	/	/	By promoting the stability of β-catenin to activate the Wnt path	Promoting the growth of colon cancer and increasing invasion	[93]
	H3K18la	/	p300	Facilitating NSUN2 expression to achieve metabolic reprogramming in an m5C-dependent manner, up-regulating glycolysis and lactate production	Promoting cell proliferation and invasive capabilities, facilitating the growth of colon cancer	[223]
	NSUN2 K356la	/		Enhancing its ability to capture target RNA, participating in the positive feedback loop regulation of the NSUN2/YBX1/m5C-ENO1 signaling axis		
	MRE11 K673la	SIRT1, SIRT2	p300/CBP	Promoting homologous recombination repair of DNA double-strand breaks	Impeding the therapeutic effects of cisplatin or PARP inhibitors (PARPi) on colorectal cancer	[224]
Non-small cell lung cancer	H3K18la	/	/	Binding to the promoter region of the POM121 gene and promoting its expression, POM121 can induce PD-L1 expression by enhancing MYC nuclear translocation	Associated with bad prognosis in non-small cell lung tumor patients, enhancing immune escapion in non-small cell lung cancer cells by inhibiting the antitumor activity of CD8^+^ T cells	[170]
	APOC2K70la	HDAC3	p300	The lactylation of APOC2 at the K70 site stabilizes protein levels by inhibiting its ubiquitination	Enhancing the release of FFA to increase Treg abundance, promoting tumor cell migration and lung metastasis in tumor tissues, and enhancing the immunotherapy resistance of NSCLC	[225]
	SOX2	/	/	Hypoxia maintains cancer cell stemness by promoting glycolysis-induced lactylation modification of SOX9	Stimulated tumor growth and promoted the growth and metastasis of cancer cells	[226]
Melanoma	H3K18la	/	/	By enhancing the expression of ALKBH3 to remove m1A methylation of SP100A, it weakens the formation of tumor-suppressive PML bodies in acute promyelocytic leukemia protein	Involved in chemotherapy resistance and promoting the malignant transformation of cancer	[227]
	H3K18la	/	p300	Facilitating the expression of YTHDF2, promoting its recognition of target mRNA to enhance the transcription of PER1/TP53	Promoting tumor cell growth and proliferation, involved in the occurrence and development of uveal melanoma	[101]
	H3K18	/	/	Reducing cell proliferation ability, promoting cellular oxidative phosphorylation to participate in metabolic reprogramming	Enhancing tumor immune suppression, promoting tumor proliferation and migration	[100]
Renal cancer	H3K18la	/	p300	Promoting the expression of PDGFRβ in response to extracellular PDGFβ signaling to enhance glycolysis for the formation of the “H3K18la/PDGFRβ” positive feedback regulation	Enhancing cellular tumorigenicity, promoting tumor growth and migration, and associated with poor prognosis in patients	[106]
	H3K14la, H3K18la, H3K56la	/	/	/	Involved in the promoting effect of FKBP10 on the proliferation, migration, and metastasis of renal clear cell carcinoma	[228]

### Targeting lactylation strategies (Fig. 5 and Table 2)

#### Gene interference

Gene therapy represents a crucial avenue in cancer treatment, encompassing strategies based on genes, RNA, and immune responses [107]. RNA interference, a form of gene interference, operates at the RNA level to silence specific genes by inhibiting their transcription or translation, thereby suppressing tumor development [108].

Under hypoxic conditions, cancer cells generate lactate via LDHA, and the target genes of c-Myc and hypoxia-inducible factor 1 (HIF-1) encode LDHA. While the precise mechanism of LDHA inhibition through RNA suppression remains elusive, studies have demonstrated that LDHA gene knockout can delay leukemia progression [109].

Lactate synthesized by cancer cells is extruded via MCTs and CD147 [110]. Genetic interference targeting MCT1 or CD147 disrupts tumor metabolism, ultimately inducing cell death [111]. MCTs as the members of the solute carrier transporter protein family are currently extensively employed in targeted treatment strategies [112]. In the context of bladder cancer, patients expressing MCT1 and CD147 exhibit a worse prognosis when only treated by platinum-based chemotherapy. RNA interference-mediated silencing of CD147 reduces MCT1 and MCT4 expression, thereby enhancing sensitivity to cisplatin [113]. Analogous phenomena have been documented in many tumors, including gliomas, gastric cancer, CRC, and other neoplasms [114–116].

**Fig. 5.**
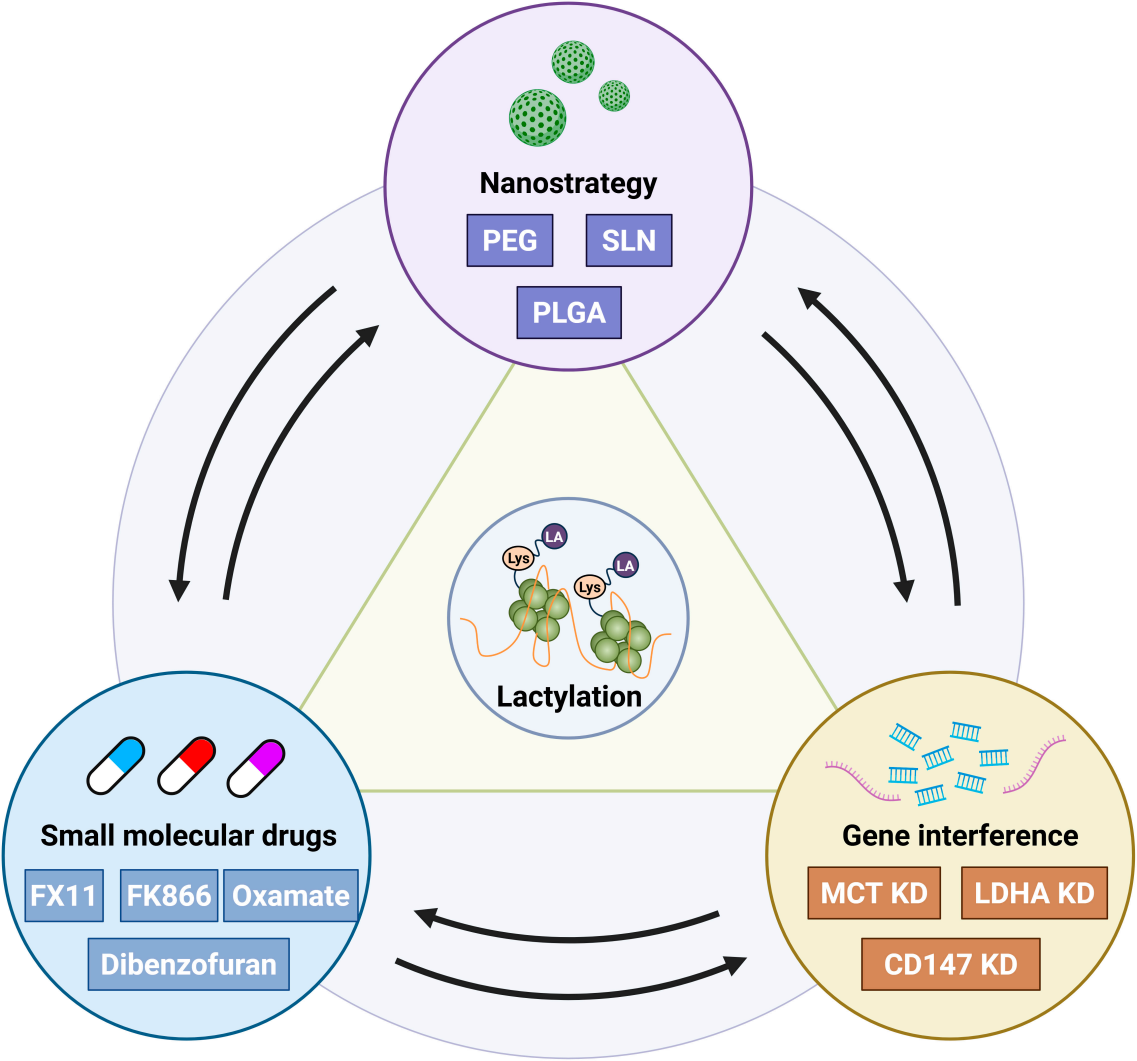
Therapeutic strategies targeting lactylation-mediated pathways in tumor. This figure delineates diverse potential intervention strategies targeting lactylation modifications, encompassing small-molecule inhibitors, genetic interference techniques, and nanotechnology-based approaches. These approaches hold promise for the development of novel lactylation-targeted interventions in future cancer therapeutics.

**Table 2.** Novel therapeutic strategies targeting lactylation

Targeting strategies	Targets	Intervention pathways/drugs	Effects on cells	Effects on cancer	References
Gene interference	LDHA	RNA interference	Reduce cell proliferation, induce cell cycle arrest at the G0/G1 phase	Delay cancer progression	[108,109]
	MCT1		Inhibit glycolysis	Disrupt tumor metabolism and promote cell death	[111,113]
	CD147		Suppress the generation of cancer stem cells	Improve survival rates and reduce tumor recurrence	[113,230]
Small molecular drugs	LDHA	FX11	Selectively induce apoptosis, promote G1 phase cell cycle arrest	Block the growth of tumor cells, demonstrating preclinical efficacy	[125]
	NAD^+^	FK866	Inhibit glycolysis by suppressing LDHA activity and affects glutamine utilization	Induce cancer regression	[125,231]
	LDH	Oxamate	Competitively inhibit LDH, promote apoptosis by enhancing mitochondrial ROS production	Inhibit tumor growth and increase sensitivity to radiotherapy	[126]
			Reduce tumor cell survival by inhibiting the Akt/mTOR pathway	Decrease cancer proliferation and control cancer progression	[127]
	Other LDH isoenzymes	Phthalimide	Inhibit LDHA and other isoenzymes, reducing lactate production and secretion	/	[128]
		Dibenzofuran			
Nanostrategy	/	PLGA carrier	Influence lactate metabolism	Enhance bioavailability and reduce drug toxicity	[131]

#### Small-molecule or clinical drug strategies

LDHA is frequently overexpressed across a broad spectrum of malignancies and is often associated with unfavorable clinical outcomes [117]. This overexpression reflects the metabolic reprogramming characteristic of many tumors, particularly those reliant on aerobic glycolysis (the Warburg effect), where LDHA plays a pivotal role in converting pyruvate to lactate and sustaining NAD^+^ regeneration.

In response to the central role of LDHA in cancer metabolism, a number of small-molecule inhibitors have been developed to target this enzyme. Among them, FX11, a selective LDHA inhibitor, has demonstrated promising antitumor activity. Notably, when used in combination with FK866, an inhibitor of NAD^+^ biosynthesis, FX11 induces significant tumor regression in preclinical lymphoma models [118]. Beyond lymphoma, FX11 has shown therapeutic potential in a variety of LDHA-dependent tumors, including colon cancer, neuroblastoma, HCC, pancreatic cancer, melanoma, and breast cancer [118–125]. These findings suggest that LDHA inhibition may be a viable strategy across multiple cancer types, especially when integrated into combination regimens targeting cancer metabolism more broadly.

Another well-characterized LDH inhibitor is oxamate, a structural analog of pyruvate that competitively inhibits the enzymatic conversion of pyruvate to lactate. Oxamate has been reported to suppress tumor cell proliferation and progression in several experimental models, including acute lymphoblastic leukemia, nasopharyngeal carcinoma, and gastric cancer cell lines [109,126,127]. Although less selective than FX11, oxamate provides important proof-of-concept evidence supporting LDH inhibition as a therapeutic approach.

More recently, novel small-molecule inhibitors with enhanced specificity for the LDHA isoform (LDH5)—such as ortho-phenylenediamine and dibenzofuran derivatives—have been identified and show improved biochemical selectivity and potency [128]. These developments highlight a key clinical imperative: the design of LDHA inhibitors with optimized selectivity, pharmacokinetic properties, and tumor-penetrating capabilities.

LDHA inhibitors offer a promising avenue for targeting the metabolic vulnerabilities of cancer cells, particularly in tumors characterized by high glycolytic flux and lactate production. Importantly, lactate not only is a metabolic byproduct but also serves as a substrate for protein lactylation. Since LDH activity directly governs intracellular and extracellular lactate levels, pharmacological inhibition of LDHA may influence the extent of protein lactylation in tumor cells. Thus, future research on LDH-targeted therapies may benefit from integrating the regulatory landscape of lactate-driven epigenetic modifications, paving the way for combined strategies that simultaneously disrupt tumor metabolism and modulate lactylation-dependent gene expression.

#### Nanoparticle strategies

Polyethylene glycol-conjugated proteins and small molecules demonstrating significant polymer–polymer interactions can function as nanoscale therapeutic agents or nanoparticles [129]. Liposomes encapsulating chemotherapeutic small-molecule drugs are utilized as solubilizing agents in cancer treatment [130].

Nanoparticle delivery systems offer a novel approach to mitigate the side effects associated with traditional drug delivery methods. Poly(lactic-co-glycolic acid) (PLGA), a biopolymer nanoparticle material, undergoes hydrolysis in vivo, yielding lactate and glycolic acid monomers that enter the citric acid cycle, thereby influencing lactate metabolism [131]. Additionally, PLGA can mitigate the side effects of chemotherapeutic agents, elevate drug concentrations, augment efficacy, and create a stable environment for drug action [132]. However, PLGA is susceptible to rapid activation by mononuclear macrophages, resulting in premature drug release and inadequate tumor acid-responsive drug release. Consequently, chitosan and polyethylene glycol are frequently employed to optimize drug encapsulation [133].

In clinical applications, PLGA-based systems are widely utilized as delivery vehicles for anticancer agents [134]. In HCC treatment, PLGA serves as a carrier for sorafenib, addressing its poor water solubility, rapid clearance, and limited absorption [135]. For prostate cancer therapy, PLGA nanoparticles are employed to encapsulate indocyanine green and docetaxel, enhancing therapeutic efficacy [136]. Additionally, the release of copper ions or copper ion carriers within the CRC microenvironment induces cuproptosis, thereby enhancing therapeutic efficacy [137]. Analogous strategies are applied in many tumors, such as breast cancer, lung cancer, and other malignancies [138,139].

**Fig. 6.**
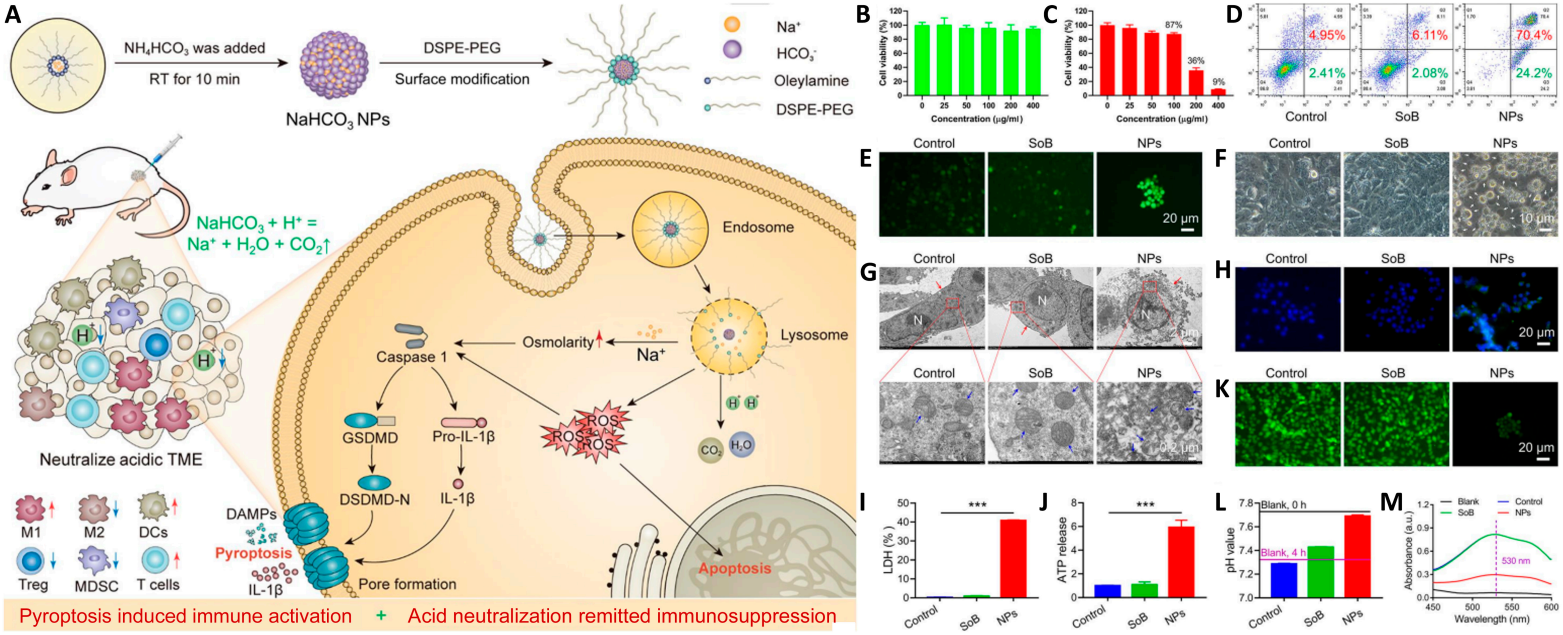
Nanoparticle-mediated immunotherapeutic approaches for modulating lactate levels in the TME. Mildly alkaline NaHCO3 nanoparticles modulate lactate metabolism through acid-base neutralization, consequently reversing the slightly acidic, immunosuppressive TME. These nanoparticles demonstrate potential as an adjuvant in tumor immunotherapy. (A) Schematic of NaHCO₃ nanoparticles designed to modulate tumor acidity and trigger pyroptosis, thereby enhancing antitumor immune responses. (B) Nanoparticle therapy attenuates tumor cell activity. (C) Nanoparticle therapy induces cellular apoptosis. (D to J) Apoptosis, ROS levels, cell morphology, ultrastructural changes, pyroptosis-related protein expression, and cellular damage indicators were evaluated in 4T1 cells following nanoparticle treatment. (K and L) Nanoparticle therapy elevates the pH of the TME and mitigates extracellular lactate release. (M) Extracellular lactic acid detection [219].

**Fig. 7.**
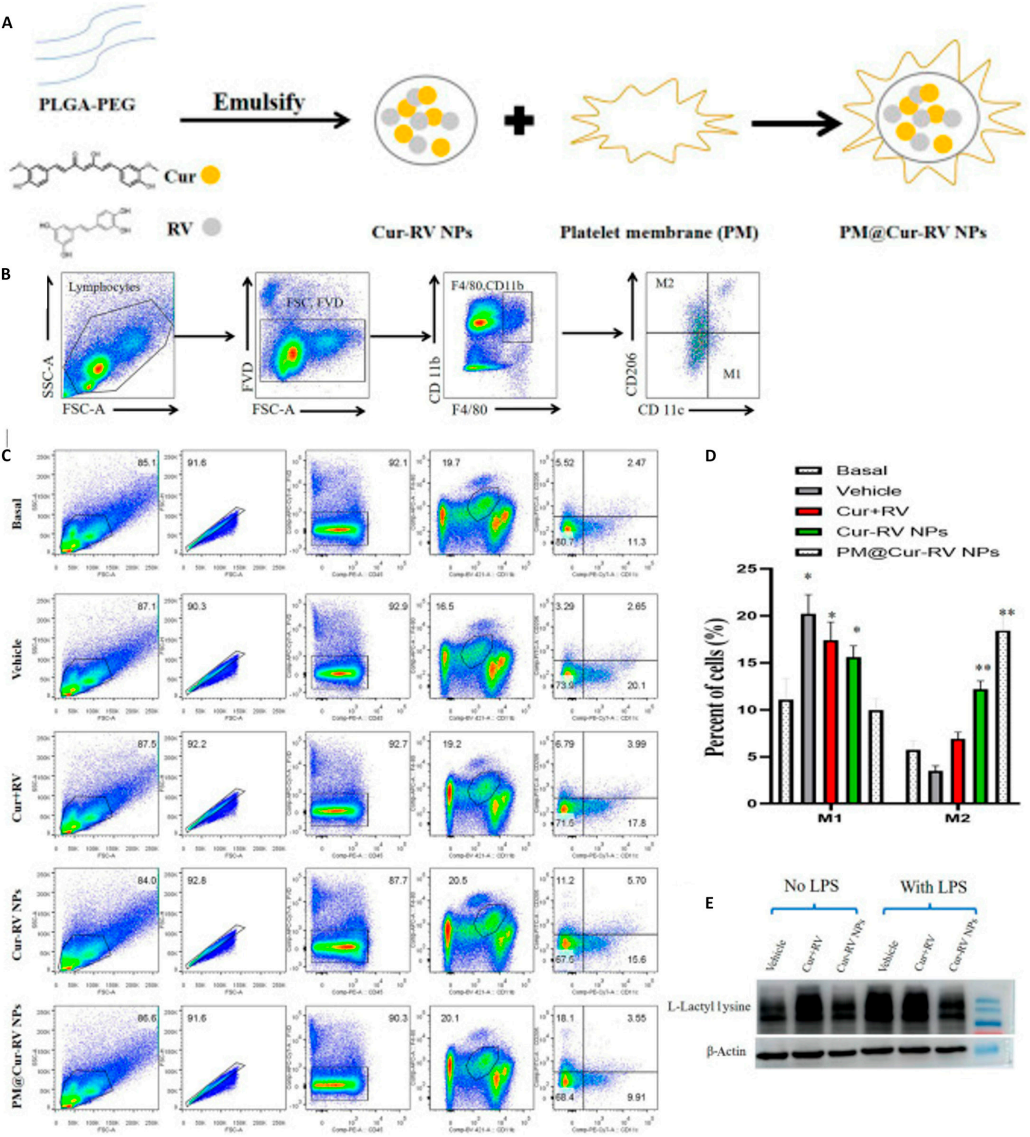
Nanoparticle-based therapeutic agents attenuate lactylation modifications in tumor. Figure demonstrates that PLGA-based anti-inflammatory nanoparticles loaded with curcumin/resveratrol attenuate lipopolysaccharide (LPS)-induced lactylation modifications. (A to E) PM@Cur-RV nanoparticles were prepared via emulsification and shown to promote M2 macrophage polarization and reduce L-lactyl lysine levels in inflammatory models, as confirmed by flow cytometry and Western blot analyses [220].

While nanoparticle technology has not yet been applied to inhibit lactate production in cancer treatment, nano-liquid chromatography–mass spectrometry (LC-MS) and LC-MS/MS technologies are frequently employed for comprehensive N-glycan analysis in murine glycosylation models [140]. LC-MS and LC-MS/MS technologies have emerged as predominant methodologies for glycomic analysis [141–144], demonstrating significant utility in diverse applications from polysaccharide biomarker discovery to glycosylated biopharmaceutical analysis [145–150]. In investigations of CARD9-related genetic immune disorders, high-precision nano-LC-MS/MS is employed for lactylation analysis, informing the development of novel clinical treatment strategies [151] (Figs. 6 and 7).

## Lactate-Induced Microenvironmental Remodeling in Tumor: Implications for Tumor Progression and Therapy Resistance

### Composition and characteristics of the TME

The TME constitutes a highly dynamic and multifaceted ecosystem that encompasses not only malignant cells but also a diverse array of nontransformed stromal and immune elements, as well as a complex repertoire of secreted signaling molecules [152]. This environment includes heterogeneous tumor cell populations, infiltrating and tissue-resident immune cells, diverse stromal subsets, soluble factors, and a structurally intricate ECM [153]. In addition to providing structural support, the ECM serves as a dynamic platform for intercellular communication, comprising critical elements such as CAFs, pro-angiogenic immune cell subsets, and vascular-associated cell types (VACs) [154]. Host-derived cellular components include CAFs, tumor endothelial cells (TECs), and a broad range of immunomodulatory populations possessing both effector and immunosuppressive properties [153]. Through persistent and bidirectional molecular and metabolic crosstalk, this cellular consortium significantly influences the metabolic adaptability and survival mechanisms of tumor cells [155].

Recent advancements in cancer immunotherapy underscore the central role of the TME in shaping therapeutic outcomes and determining clinical prognosis [156]. Once viewed as a passive scaffold, the TME is now recognized as an active player in tumor evolution—an interactive domain where neoplastic, stromal, and immune compartments dynamically coevolve and reprogram one another. A key defining feature of this ecosystem is the tumor’s immunological landscape, which is often categorized into “immune-hot” and “immune-cold” phenotypes. Immune-hot tumors are characterized by dense cytotoxic lymphocyte infiltration and heightened pro-inflammatory signaling, making them more responsive to immune checkpoint blockade. Conversely, immune-cold tumors exhibit limited immune cell infiltration and a tolerogenic microenvironment, which is frequently associated with resistance to immunotherapeutic strategies [157,158]. This immunological dichotomy is closely intertwined with tumor metabolic reprogramming, particularly the Warburg effect—a hallmark metabolic shift wherein cancer cells favor aerobic glycolysis even under normoxic conditions [25]. This metabolic shift leads to substantial accumulation of intracellular and extracellular lactate—the ionized form of lactic acid at physiological ph. Far from a mere metabolic byproduct, lactate has emerged as a potent immunometabolic regulator [159,160]. It acidifies the extracellular milieu, dampens the cytotoxic functions of effector T and NK cells, and promotes an immunosuppressive niche conducive to tumor progression. As previously noted, lactate also acts as a substrate for histone lactylation—a recently discovered epigenetic mark capable of reprogramming transcriptional landscapes in tumor and immune cells alike. Such lactylation-dependent regulation has been implicated in promoting T cell exhaustion, macrophage polarization, and Treg stability—mechanisms that synergistically contribute to immune escape [161–164].

**Fig. 8.**
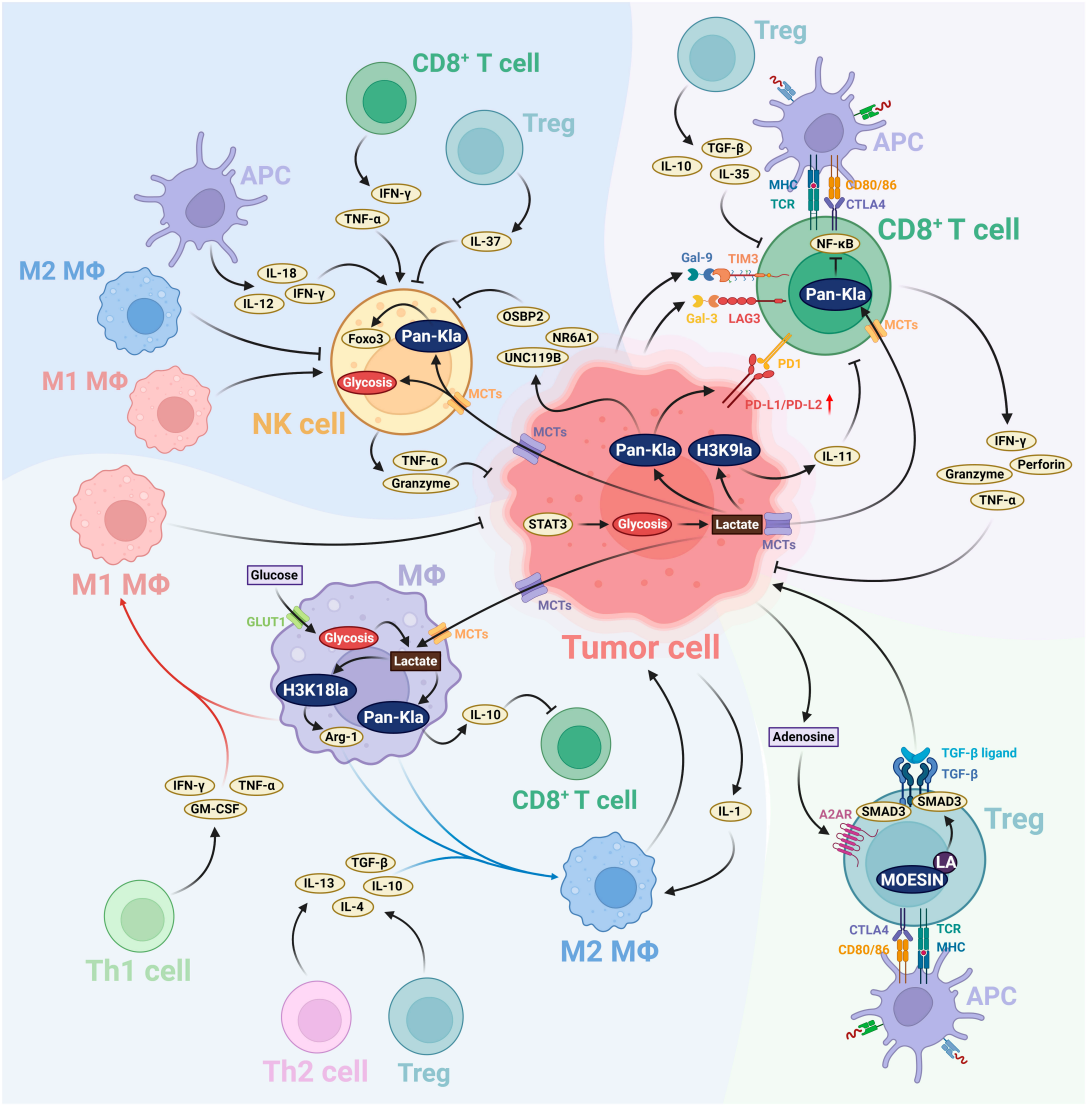
Multifaceted roles of lactylation in modulating the TME. Histone and nonhistone lactylation modifications exert critical influences on diverse immune cell populations within the TME. A substantial body of evidence indicates that lactate, produced or secreted by neoplastic cells, exhibits multifaceted effects: suppressing the cytotoxic activities of various immune cells, promoting tumor proliferation, compromising therapeutic efficacy, and facilitating metastatic progression (Table 1).

Therefore, the Warburg effect contributes not only to energy metabolism but also to immunomodulation via lactylation. In the subsequent sections, we explore how this integrated metabolic–epigenetic axis modulates the activity of critical immune cell subsets—including CD8^+^ T cells, NK cells, TIMs, and Tregs—providing a framework for therapeutic strategies aimed at targeting lactate metabolism and lactylation (Fig. 8).

#### CD8^+^ T cells

As key mediators of antitumor immunity, CD8^+^ cytotoxic T lymphocytes (CTLs) are essential for recognizing and eliminating malignant cells. Their effector function is primarily driven by the release of cytotoxic granules (perforin, granzyme B) and proinflammatory cytokines, including IFN-γ and tumor necrosis factor-α (TNF-α) [165,166]. However, in the immunosuppressive TME, CD8^+^ T cell cytotoxicity is often impaired, facilitating immune evasion and tumor progression. The TME imposes diverse metabolic and molecular constraints that influence T cell differentiation and effector function [167,168]. Through aerobic glycolysis, tumor cells consume large quantities of glucose and secrete excess lactate, leading to a nutrient-deficient and acidic microenvironment. This metabolic competition deprives CD8^+^ T cells of critical nutrients—particularly glucose and amino acids—thereby limiting their effector capacity and proliferation [168]. Simultaneously, lactate accumulation remodels the epigenetic landscape of both tumor and immune cells via histone lactylation [163,169]. Recent evidence shows that specific lactylation including H3K9la and H3K18la exert distinct temporal and functional effects on gene regulation in CD8^+^ T cells. In quiescent CD8^+^ T cells, H3K9la is preferentially enriched at genes associated with cellular dormancy and promotes oxidative phosphorylation. By contrast, during activation, H3K18la accumulates at effector gene loci, promoting glycolytic reprogramming and enhancing immune responses [163]. PD-L1, a key immunotherapy target, is subject to regulation by lactylation. Recent findings suggest that lactate-induced histone lactylation in tumor cells indirectly suppresses CD8^+^ T cell responses by up-regulating immune checkpoints such as PD-L1 through STAT5-mediated glycolytic activation [170,171]. Moreover, H3K18la enrichment in tumor cells increases B7-H3 expression, reducing CD8^+^ T cell infiltration and promoting resistance to PD-1 blockade [169]. Beyond transcriptional regulation, lactylation also affects PD-L1 protein stability via nongenomic mechanisms. A recent study found that limiting serine and glycine availability enhances PD-L1 lactylation, stabilizing the protein by preventing its degradation [172]. This uncovers a novel mechanism whereby metabolic cues stabilize immune checkpoint proteins independently of gene transcription, potentially affecting immunotherapy outcomes. Additionally, H3K9la-dependent IL-11 signaling contributes to CD8^+^ T cell exhaustion, a dysfunctional state marked by reduced cytokine secretion [173]. Other studies indicate that histone lactylation may regulate sensitivity to activation-induced cell death (AICD) via suppression of NF-κB signaling [174]. Although PD-L1–mediated suppression is well documented, the broader roles of lactylation in regulating CD8^+^ T cell function via other ligand–receptor pathways remain largely unexplored. Since exhausted T cells pose a key challenge to immunotherapy, elucidating how lactate metabolism and lactylation shape CD8^+^ T cell fate may enable the development of novel therapeutic strategies.

In summary, metabolic remodeling within the TME profoundly shapes the phenotype, function, and therapeutic responsiveness of CD8^+^ T cells. Lactate buildup and histone lactylation have emerged as central regulators of T cell exhaustion and tumor immune evasion. Deciphering these mechanisms not only deepens our understanding of TME-mediated immunosuppression but also paves the way for metabolism-oriented immunotherapies in cancer.

#### NK cells

NK cells serve as essential early responders within the innate immune system, offering frontline protection against virally infected and malignant cells. In cancer, NK cells are particularly significant for their ability to detect and eliminate circulating tumor cells, thereby restricting metastatic spread [175,176]. Accordingly, NK cell functionality is now recognized as a critical determinant of disease progression and response to therapy. However, in the immunosuppressive TME, NK cell activity is significantly impaired. This dysfunction is primarily driven by metabolic stressors, including nutrient deprivation (e.g., glucose, glutamine, and oxygen) and the buildup of immunosuppressive metabolites such as adenosine and lactate [177]. These metabolic constraints not only impair NK cell cytotoxicity but also affect their infiltration, survival, and cytokine secretion [178]. Recent clinical studies have revealed a strong association between elevated intratumoral lactate levels and diminished NK cell infiltration, with high “lactate scores” acting as negative prognostic markers in various cancers. Transcriptomic profiling has identified up-regulation of genes such as NR6A1, OSBP2, and UNC119B, which are believed to mediate lactate-driven suppression of NK cell recruitment. These findings indicate that lactate functions not just as a metabolic byproduct, but as an active regulator of immune exclusion within the TME [179,180]. Supporting this concept, natural killer T (NKT) cells isolated from malignant pleural effusions demonstrate increased lactate uptake and utilization, reflecting metabolic adaptation to a high-lactate environment [181]. Although direct evidence for lactylation-mediated dysfunction in NK cells is limited, parallels with CD8^+^ T cell biology suggest that similar regulatory mechanisms may be involved.

#### TIM cells

TIMs have an important impact in tumor immune evasion. Conventionally, macrophage activation states are categorized into 2 main types: the promoting inflammatory and cytotoxic M1 phenotype, and the reducing inflammatory and reparative M2 phenotype. The M2 phenotype is associated with reduced inflammatory infiltration, enhanced angiogenesis, and maintenance of an immunosuppressive TME, factors linked to poor prognosis and tumor metastasis [182,183]. While lactate has been demonstrated to promote TIM polarization toward the M2 phenotype in various tumors within the TME, its precise mechanism of action remains elusive [184–186].The 2019 discovery of lactate modification has established a direct link between epigenetic regulation and metabolic reprogramming. Studies revealed that H3K18 lactylation (H3K18la) promotes the demonstration of M2-like genes, such as Arg-1, facilitating macrophage polarization and phenotypic alteration [7]. Subsequent investigations in prostate cancer have demonstrated that modulating lactate levels can influence tumor progression through H3K18la-mediated mechanisms [187]. In glioma cells, GLUT1-derived lactate enhances histone lactylation in TIMs, promoting IL-10 transcription and potentially suppressing T cell activity [188]. Similarly, in colon cancer, tumor-derived lactate up-regulates H3K18la in TIMs, inhibiting RARγ transcription and fostering tumor development [189]. Intriguingly, lactylation of the nonhistone protein HMGB1 has been implicated in M1 macrophage polarization during sepsis [190]. Microglia, as the resident mononuclear phagocytes of the central nervous system, exhibit increased neuroinflammation and aging-related secretory phenotypes when H3K18 lactylation (H3K18la) levels are elevated. Concurrently, H4K12 lactylation (H4K12la) enhances the expression of PKM2, a key glycolysis gene, thereby augmenting lactate production. This creates a “H4K12la-PKM2” positive feedback loop, potentially contributing to the progression of AD [191,192].

These findings appear to contradict the traditional anti-inflammatory role ascribed to lactate in the TME, independent of tissue and cell specificity. We propose 2 potential explanations for this apparent discrepancy. Lactylation has 2 isomeric forms: l-lactylation and d-lactylation. l-Lactate predominates in human and eukaryotic systems. l-Lactylation, the most extensively studied form, has been involved in various pathological processes, including tumorigenesis, cardiovascular diseases, neurological disorders, and gastrointestinal pathologies [7,193–195]. In contrast, d-lactylation, a recently identified modification, presents a vast unexplored research frontier. Studies have shown that d-lactate accumulation can induce acute neurotoxicity [196]. Furthermore, d-lactate derived from the intestinal microbiota can alter the morphology and size of hepatic Kupffer cells via portal circulation, enhancing their pathogen capture and elimination capabilities [197]. These findings may elucidate the seemingly paradoxical role of lactate in promoting neuroinflammation and M1 macrophage polarization. Lactylation may be considered a consequence rather than a cause of cellular metabolic states. M1-polarized macrophages predominantly rely on glycolysis to produce energy, while M2-polarized macrophages primarily utilize fatty acid oxidation and oxidative phosphorylation. The metabolic divergence between these phenotypes may contribute to differences in lactate availability and lactylation levels [198]. Consequently, M1 polarization is associated with increased lactate production and lactylation modifications. This presents an apparent paradox: Elevated lactylation during M1 polarization potentially facilitates the transition to M2 polarization. This seeming contradiction has been addressed in seminal work on lactylation modification through the introduction of the “lactate clock” concept. This model elucidates the temporal regulation of lactate on polarization balance. Notably, lactylation modification exerts its regulatory influence predominantly during the later stages of M1 polarization, accounting for the observed phenomenon [7]. In light of these findings, we posit that lactylation modification is more likely a consequence rather than an initiating factor in many physiological and pathological process.

#### Treg cells

Regulatory T cells (Tregs) constitute an immunosuppressive subset of CD4^+^ T cells that are often highly enriched in the TME. They maintain immunosuppressive conditions by expressing inhibitory receptors (e.g., CTLA-4 and PD-1) and secreting anti-inflammatory cytokines such as IL-10 and TGF-β. High levels of Treg infiltration are frequently linked to poor clinical prognosis and reduced responsiveness to immune checkpoint blockade therapies [199]. Recent studies indicate that the immunosuppressive function of tumor-infiltrating Tregs is closely tied to their capacity to metabolize lactate as a fuel source [200]. In the nutrient-deprived and lactate-rich TME, Tregs display increased metabolic plasticity, enabling them to survive in conditions that inhibit conventional effector T cells [200,201]. Notably, lactate uptake facilitates epigenetic modifications of key regulatory proteins, thereby strengthening Treg-mediated immunosuppression. One notable mechanism involves the lactylation of moesin, a cytoskeletal-associated nonhistone protein. In Tregs, lactylated moesin activates the TGF-β/SMAD3 signaling cascade, thereby driving tumor progression in murine models [202]. This finding highlights lactate’s dual role as both an energy substrate and a signaling metabolite capable of modulating immunosuppressive pathways. Interestingly, LDHA inhibition via oxamate paradoxically increases Treg infiltration in glioblastoma [203], suggesting that lactate metabolism exerts complex, context-dependent effects on Treg behavior. These findings underscore the need for deeper mechanistic insights into how lactate and its downstream modifications regulate Treg function across diverse tumor contexts.

**Fig. 9.**
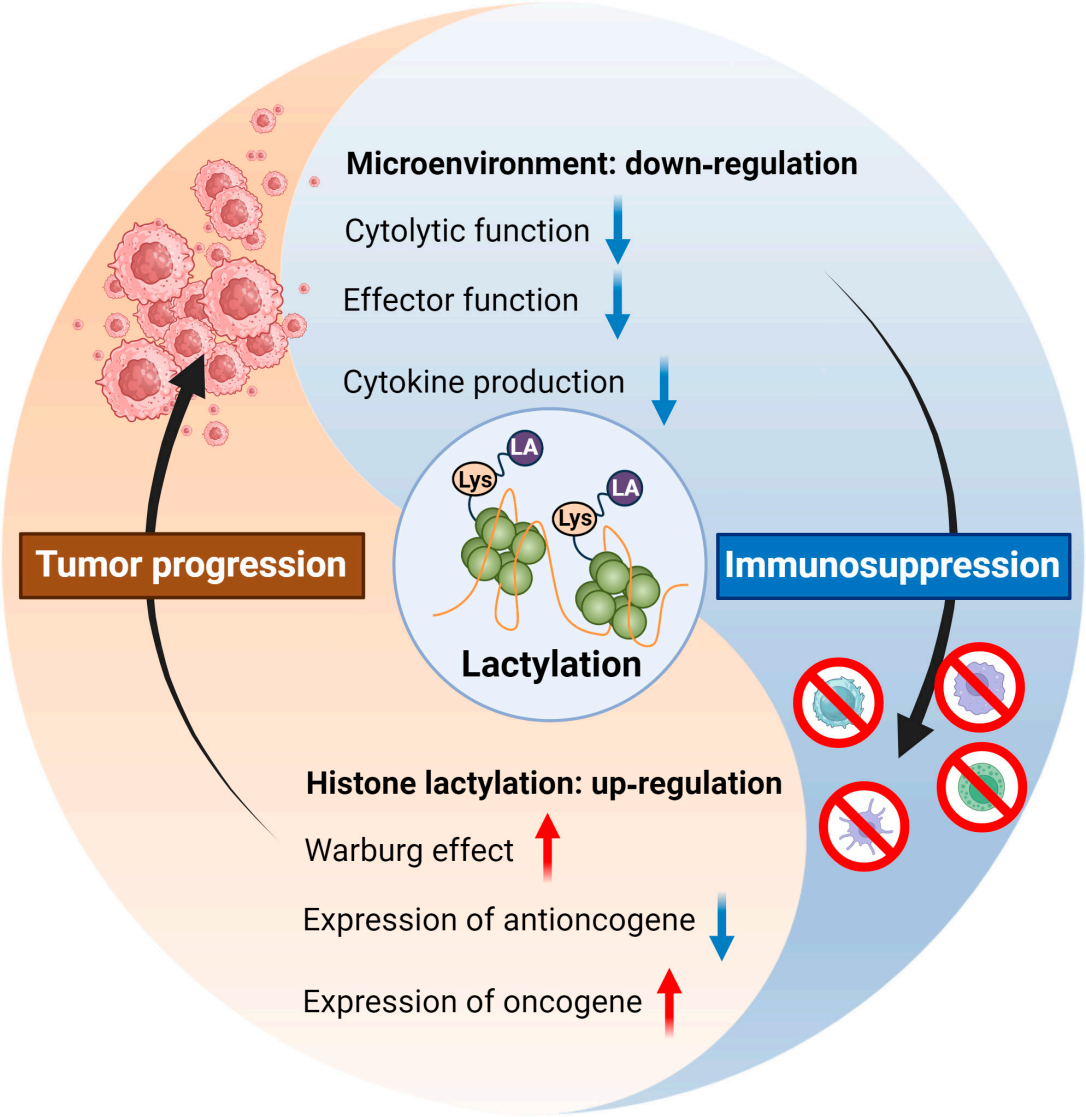
Influence of lactylation on tumor progression and its TME dynamics. In neoplastic tissues, lactylation modifications promote malignant progression through multiple mechanisms, including enhanced glycolysis, oncogene activation, and tumor suppressor gene repression. Within the TME, lactylation modifications attenuate the immune emission of cytotoxic CD8^+^ T cells, tumor-infiltrating macrophages (TIMs), and NK cells, thereby compromising antitumor immune responses. Conversely, these modifications augment Treg activity, further facilitating the establishment of an immunosuppressive TME and consequently exacerbating immune evasion.

In conclusion, both histone and nonhistone lactylation modifications exert significant effects on various immune cells within the TME, including NK cells, CD8^+^ T cells, TIMs, and Tregs. These modifications regulate key tumor-related processes, including immune evasion and metastasis, and are closely associated with patient prognosis and overall survival. Consequently, targeting lactate metabolism or lactylation modifications represents a promising strategy for the clinical management of various malignancies. Although de-lactylase enzymes have been identified in various tumor cells and shown to modulate lactylation status and biological processes (Table 3), their roles in TME-resident immune cells and corresponding effects on lactylation dynamics and cellular function remain poorly understood. As a result, Table 1 provides only putative de-lactylase enzymes at specific modification sites. Further direct evidence is needed to clarify the roles of de-lactylase enzymes in TME immune cells and their impact on tumor biology. Defining the functional roles and underlying mechanisms of lactylation modifications within the TME will establish a strong conceptual foundation for cancer therapy. Such insights may broaden therapeutic strategies in oncology and inform the treatment of nonmalignant diseases. Future research focusing on the complex interplay between lactylation, immune cell function, and tumor progression will be critical for translating these findings into clinically applicable therapies (Fig. 9).

**Table 3.** Lactylation-induced immunomodulation in the tumor microenvironment: Effects on immune cell function and antitumor responses

TME immune cells	Lactylation modification site	Potential delactylation enzyme	Lactyl transferase	Protein modification effects/target genes	The impact on tumors	Cite
CD8^+^ T cell	H4K5la	HDAC1, HDAC3	E3BP	Enriched in the PD-L1 promoter region to promote its expression	Activating PD-L1/PD-1, causing a decrease in CD8^+^ T cell activity, leading to evasion and reducing the survival period of AML patients	[57,171]
	H3K9la	HDAC2	/	Promoting the expression of IL-11, which activates immune checkpoint gene transcription by stimulating the JAK2/STAT3 signaling pathway	Participating in lactate-induced exhaustion of CD8^+^ T cells, leading to immune escape and resistance to immunotherapy in head and neck squamous cell carcinoma	[173,229]
	H3K18la	HDAC1/2/3, SIRT1/2/3	p300	Promoting the transcription of circular RNA circATXN7 to inhibit the NF-κB signaling pathway	Inhibiting the therapeutic effect of anti-PD-1 antibodies on cancer, increasing resistance to tumor immunotherapy	[57,174]
NK cell	Pan-Kla	HDAC1/2/3/8, SIRT1/2/3	/	Associated with the expression of NR6A1, OSBP2, and UNC119B	Inhibiting NK cell infiltration, promoting tumor resistance to chemotherapy and immunotherapy, and associated with low survival rates in patients	[57,180]
TIM cell	H3K18la	HDAC1/2/3, SIRT1/2/3	p300	Promoting the expression of M2-like genes such as Arg-1, involved in regulating macrophage polarization homeostasis	/	[7,57]
	H3K18	HDAC1/2/3, SIRT1/2/3	/	Activating the Wnt/β-catenin pathway, further promoting lactate production and secretion, inhibiting TIM activation to reduce phagocytic function	Reducing the therapeutic efficacy of PI3Ki and MEKi chemotherapy on prostate cancer	[57,187]
	Pan-Kla	HDAC1/2/3/8, SIRT1/2/3	p300	Enriched in the IL-10 promoter region, regulating its expression and secretion	Inducing T cell exhaustion within tumors, promoting tumor growth and progression of glioblastoma	[57,188]
	H3K18la	HDAC1/2/3, SIRT1/2/3	p300	Inhibiting RARγ transcription to suppress the TRAF6/NF-κB/IL-6/STAT3 signaling pathway	Reducing the antitumor function of macrophages, involved in the treatment of colorectal cancer with nordihydroguaiaretic acid	[57,189]
Treg cell	MOESIN K72	/	/	Facilitating the interaction between MOESIN and TGF-βR1 to enhance the TGF-β/SMAD/FOXP3 signaling pathway, participating in the development and function of Treg cells	Promoting tumorigenesis, inhibiting the therapeutic effect of anti-PD-1 immunotherapy on hepatocellular carcinoma	[202]

### Writer, reader, and eraser

The enzymatic pathway of lactylation modification requires 3 key components: writers, readers, and erasers, which respectively insert, recognize, and remove lactylation modifications on histones. Recent studies have elucidated that the acetyltransferase p300 functions as a writer in this process, utilizing lactate as a substrate to catalyze lactylation [57,204]. The most prevalent erasers identified thus far are HDAC1 to HDAC3, which exhibit robust de-modification activity toward both d-lactate and l-lactate isomers. Although these enzymes demonstrate slight preferences, such as enhanced de-modification activity toward d-lactate, they generally show minimal sensitivity to the chiral center configuration of lactate [57]. In the context of HCC, an additional eraser has been identified: the NAD^+^-dependent deacetylase SIRT3. This enzyme has been demonstrated to inhibit the growth-promoting effects of cell cycle protein E2 on liver cancer cells, presenting a promising avenue for potential therapeutic interventions in HCC [205].

## Summary and Outlook

Lactylation is a posttranslational histone modification, analogous to acetylation, methylation, and phosphorylation. Lactylation contributes to inflammatory responses through multiple mechanisms, including the regulation of macrophage phenotype transitions [206] and modulation of T helper 17 (Th17) cell metabolism and epigenetic states [207], and activation of NF-κB signaling pathway in central nervous system may promote aging and accelerate the progress of AD [191,192]. Within the cardiovascular system, lactylation has been demonstrated to exert contrary inflammatory effects and promote angiogenesis, thereby enhancing cardiac function following myocardial infarction [208,209]. Furthermore, lactic acidification plays a crucial role in the inflammatory response associated with intervertebral disc degeneration [210].The reduction of lactate levels associated with LCP1 following cerebral infarction has been demonstrated to slow disease progression. Cerebral ischemia–reperfusion injury demonstrates a strong correlation with calcium overload in neural tissues. Lactylation of key proteins in calcium channels induces mitochondrial damage, subsequently compromising neuronal activity and ultimately resulting in neuronal death. These findings suggest that lactate exerts deleterious effects in cerebral infarction, thereby presenting potential therapeutic targets for post-infarction treatment strategies [211,212]. Lactylation has also been implicated in various respiratory disorders, including pulmonary fibrosis and pulmonary arterial hypertension [213,214]. Accumulating evidence proves that lactylation has a pervasive role in diverse pathophysiological processes, with relevance to tumor formation, invasion, and immune evasion. Consequently, modulation of lactylation represents a promising avenue for therapeutic intervention in oncology (Fig. 1).

Recent studies have revealed that lactylation occurs on nonhistone proteins and even noncoding RNA in plants and microorganisms. However, further investigation is warranted to elucidate if this phenomenon is present in human systems [215]. Moreover, d-lactylation modification is ubiquitous in human cells, yet it exhibits distinct characteristics from l-lactate regarding target proteins and functions [58]. Emerging evidence suggests that these structural isomeric differences may contribute to the seemingly contradictory pro-inflammatory effects of lactylation in the nervous system juxtaposed with anti-inflammatory effects in other pathological conditions. As previously discussed, lactylation modification appears to be a consequence rather than an initiating factor, with the “lactate clock” exerting regulatory control and giving rise to bidirectional effects of lactylation modification. Furthermore, recent findings indicate that RNA methylation and histone/DNA epigenetic mechanisms exhibit reciprocal influence, resulting in a cascade of effects on transcriptional output, translation, and the recruitment of chromatin modification factors [216]. These complex interactions necessitate comprehensive investigation to mitigate potential adverse effects in the development of targeted therapies.

Contemporary research predominantly focuses on the effects of lactylation in the TME, with limited exploration of specific lactylation sites. Several critical questions remain inadequately addressed, including the precise role of lactylation in tumor cell metabolism, its differential effects on diverse immune cell populations, the identification and characterization of lactylation readers, and the varied functions of lactylation across different tumor types. Elucidating these aspects will yield novel strategies for leveraging lactylation in cancer therapeutics and facilitate the development of more precise, tumor-specific targeted therapies. Research has demonstrated that in lung cancer patients treated with paclitaxel, the acidic TME may induce drug resistance by promoting drug sequestration [217,218]. Consequently, overcoming lactate-induced drug resistance represents a critical challenge in current clinical oncology. Furthermore, the integration of targeted therapies for lactylation-related metabolic processes with emerging technologies, such as nanotechnology, warrants further refinement. Undeniably, other reviews have also summarized and analyzed the role of lactylation modifications in tumors and the tumor immune microenvironment. However, this review distinguishes itself by incorporating numerous recent references, particularly focusing on d-lactylation modifications. Furthermore, in the clinical context, we have specifically and comprehensively analyzed small molecules and clinical drug approaches as well as nanoparticle-based therapies, which sets this review apart from others.

In conclusion, bolstering fundamental research on lactate metabolism and lactylation is imperative to establish new directions and a robust theoretical foundation for clinical interventions. Targeting lactylation and developing lactylation-related immunotherapies represent promising frontiers in cancer treatment modalities.

## Data Availability

Data sharing is not applicable to this article as no datasets were generated or analyzed during the current study.

## References

[1] Chen YJ, Mahieu NG, Huang X, Singh M, Crawford PA, Johnson SL, Gross RW, Schaefer J, Patti GJ. Lactate metabolism is associated with mammalian mitochondria. Nat Chem Biol. 2016;12(11):937–943. 27618187 10.1038/nchembio.2172 PMC5069139

[2] Ferguson BS, Rogatzki MJ, Goodwin ML, Kane DA, Rightmire Z, Gladden LB. Lactate metabolism: Historical context, prior misinterpretations, and current understanding. Eur J Appl Physiol. 2018;118(4):691–728. 29322250 10.1007/s00421-017-3795-6

[3] Brooks GA. The science and translation of lactate shuttle theory. Cell Metab. 2018;27(4):757–785. 29617642 10.1016/j.cmet.2018.03.008

[4] Brooks GA. Lactate shuttles in nature. Biochem Soc Trans. 2002;30(2):258–264. 12023861 10.1042/

[5] Liberti MV, Locasale JW. The Warburg effect: How does it benefit cancer cells? Trends Biochem Sci. 2016;41(3):211–218. 26778478 10.1016/j.tibs.2015.12.001 PMC4783224

[6] Schwartz L, Supuran CT, Alfarouk KO. The Warburg effect and the hallmarks of cancer. Anti Cancer Agents Med Chem. 2017;17(2):164–170. 10.2174/1871520616666161031143301 27804847

[7] Zhang D, Tang Z, Huang H, Zhou G, Cui C, Weng Y, Liu W, Kim S, Lee S, Perez-Neut M, et al. Metabolic regulation of gene expression by histone lactylation. Nature. 2019;574(7779):575–580. 31645732 10.1038/s41586-019-1678-1 PMC6818755

[8] Fantin VR, St-Pierre J, Leder P. Attenuation of LDH-A expression uncovers a link between glycolysis, mitochondrial physiology, and tumor maintenance. Cancer Cell. 2006;9(6):425–434. 16766262 10.1016/j.ccr.2006.04.023

[9] Jha MK, Lee IK, Suk K. Metabolic reprogramming by the pyruvate dehydrogenase kinase-lactic acid axis: Linking metabolism and diverse neuropathophysiologies. Neurosci Biobehav Rev. 2016;68:1–19. 27179453 10.1016/j.neubiorev.2016.05.006

[10] DeBerardinis RJ, Mancuso A, Daikhin E, Nissim I, Yudkoff M, Wehrli S, Thompson CB. Beyond aerobic glycolysis: Transformed cells can engage in glutamine metabolism that exceeds the requirement for protein and nucleotide synthesis. Proc Natl Acad Sci USA. 2007;104(49):19345–19350. 18032601 10.1073/pnas.0709747104 PMC2148292

[11] Flick MJ, Konieczny SF. Identification of putative mammalian D-lactate dehydrogenase enzymes. Biochem Biophys Res Commun. 2002;295(4):910–916. 12127981 10.1016/s0006-291x(02)00768-4

[12] Pasti AP, Rossi V, Di Stefano G, Brigotti M, Hochkoeppler A. Human lactate dehydrogenase A undergoes allosteric transitions under pH conditions inducing the dissociation of the tetrameric enzyme. Biosci Rep. 2022;42(1): Article BSR20212654. 35048959 10.1042/BSR20212654 PMC8799922

[13] de Bari L, Atlante A, Guaragnella N, Principato G, Passarella S. D-Lactate transport and metabolism in rat liver mitochondria. Biochem J. 2002;365(Pt 2):391–403. 11955284 10.1042/BJ20020139 PMC1222695

[14] Adeva-Andany M, López-Ojén M, Funcasta-Calderón R, Ameneiros-Rodríguez E, Donapetry-García C, Vila-Altesor M, Rodríguez-Seijas J. Comprehensive review on lactate metabolism in human health. Mitochondrion. 2014;17:76–100. 24929216 10.1016/j.mito.2014.05.007

[15] Ewaschuk JB, Naylor JM, Zello GA. D-lactate in human and ruminant metabolism. J Nutr. 2005;135(7):1619–1625. 15987839 10.1093/jn/135.7.1619

[16] Scheijen JL, Hanssen NM, van de Waarenburg MP, Jonkers DM, Stehouwer CD, Schalkwijk CG. L(+) and D(-) lactate are increased in plasma and urine samples of type 2 diabetes as measured by a simultaneous quantification of L(+) and D(-) lactate by reversed-phase liquid chromatography tandem mass spectrometry. Exp Diabetes Res. 2012;2012: Article 234812. 22474418 10.1155/2012/234812 PMC3310144

[17] de Bari L, Atlante A, Armeni T, Kalapos MP. Synthesis and metabolism of methylglyoxal, S-D-lactoylglutathione and D-lactate in cancer and Alzheimer’s disease. Exploring the crossroad of eternal youth and premature aging. Ageing Res Rev 2019;53: Article 100915. 31173890 10.1016/j.arr.2019.100915

[18] Rabinowitz JD, Enerbäck S. Lactate: The ugly duckling of energy metabolism. Nat Metab. 2020;2(7):566–571. 32694798 10.1038/s42255-020-0243-4 PMC7983055

[19] Dienel GA. Brain glucose metabolism: Integration of energetics with function. Physiol Rev. 2019;99(1):949–1045. 30565508 10.1152/physrev.00062.2017

[20] Lhomme T, Clasadonte J, Imbernon M, Fernandois D, Sauve F, Caron E, da Silva LN, Heras V, Martinez-Corral I, Mueller-Fielitz H, et al. Tanycytic networks mediate energy balance by feeding lactate to glucose-insensitive POMC neurons. J Clin Invest. 2021;131(18): Article e140521. 34324439 10.1172/JCI140521 PMC8439611

[21] Daw CC, Ramachandran K, Enslow BT, Maity S, Bursic B, Novello MJ, Rubannelsonkumar CS, Mashal AH, Ravichandran J, Bakewell TM, et al. Lactate elicits ER-mitochondrial Mg2+ dynamics to integrate cellular metabolism. Cell. 2020;183(2):474–489.e17. 33035451 10.1016/j.cell.2020.08.049 PMC7572828

[22] Su W, Ye Z, Liu J, Deng K, Liu J, Zhu H, Duan L, Shi C, Wang L, Zhao Y, et al. Single-cell and spatial transcriptome analyses reveal tumor heterogeneity and immune remodeling involved in pituitary neuroendocrine tumor progression. Nat Commun. 2025;16(1):5007. 40442104 10.1038/s41467-025-60028-5 PMC12122724

[23] Chappell JC, Payne LB, Rathmell WK. Hypoxia, angiogenesis, and metabolism in the hereditary kidney cancers. J Clin Invest. 2019;129(2):442–451. 30614813 10.1172/JCI120855 PMC6355237

[24] Li X, Yang Y, Zhang B, Lin X, Fu X, An Y, Zou Y, Wang JX, Wang Z, Yu T. Lactate metabolism in human health and disease. Signal Transduct Target Ther. 2022;7(1):305. 36050306 10.1038/s41392-022-01151-3 PMC9434547

[25] Wang Y, Patti GJ. The Warburg effect: A signature of mitochondrial overload. Trends Cell Biol. 2023;33(12):1014–1020. 37117116 10.1016/j.tcb.2023.03.013 PMC10600323

[26] Xia Z, De Wever O. The plasticity of cancer-associated fibroblasts. Trends Cancer. 2025;S2405-8033(25):00108–00106. 10.1016/j.trecan.2025.04.012 40473532

[27] Fiaschi T, Marini A, Giannoni E, Taddei ML, Gandellini P, De Donatis A, Lanciotti M, Serni S, Cirri P, Chiarugi P. Reciprocal metabolic reprogramming through lactate shuttle coordinately influences tumor-stroma interplay. Cancer Res. 2012;72(19):5130–5140. 22850421 10.1158/0008-5472.CAN-12-1949

[28] Liu ZY, Wu CY, Wu RQ, Wang JC, Huang CX, Wang XY, Zhang Y, Zheng L, Chen Y, Lao XM, et al. Efflux of N1-acetylspermidine from hepatoma fosters macrophage-mediated immune suppression to dampen immunotherapeutic efficacy. Immunity. 2025;58(6):1572–1585.e10. 40460833 10.1016/j.immuni.2025.05.006

[29] Zhao Y, Li M, Yao X, Fei Y, Lin Z, Li Z, Cai K, Zhao Y, Luo Z. HCAR1/MCT1 regulates tumor ferroptosis through the lactate-mediated AMPK-SCD1 activity and its therapeutic implications. Cell Rep. 2020;33(10): Article 108487. 33296645 10.1016/j.celrep.2020.108487

[30] Ma J, To SKY, Fung KSW, Wang K, Zhang J, Ngan AHW, Yung S, Chan TM, Wong CCL, Ip PPC, et al. P-cadherin mechanoactivates tumor-mesothelium metabolic coupling to promote ovarian cancer metastasis. Cell Rep. 2025;44(1): Article 115096. 39700008 10.1016/j.celrep.2024.115096

[31] de Kivit S, Mensink M, Kostidis S, Derks RJE, Zaal EA, Heijink M, Verleng LJ, de Vries E, Schrama E, Blomberg N, et al. Immune suppression by human thymus-derived effector Tregs relies on glucose/lactate-fueled fatty acid synthesis. Cell Rep. 2024;43(9): Article 114681. 39180751 10.1016/j.celrep.2024.114681

[32] Wan N, Wang N, Yu S, Zhang H, Tang S, Wang D, Lu W, Li H, Delafield DG, Kong Y, et al. Cyclic immonium ion of lactyllysine reveals widespread lactylation in the human proteome. Nat Methods. 2022;19(7):854–864. 35761067 10.1038/s41592-022-01523-1

[33] Lu J, Li GH, Hu J, Wang Z. Genetic insights support PARP1 as a mediator in the protective association of ATP-citrate lyase inhibitors with melanoma. Commun Biol. 2025;8(1):777. 40399559 10.1038/s42003-025-07860-z PMC12095509

[34] Lai J, Liu S, Chen Y, Chen J, Li J, Liang Z, Mei X, Feng Y, Han Z, Jiang F, et al. Depletion of acetyl-CoA carboxylase 1 facilitates epithelial-mesenchymal transition in prostate cancer cells by activating the MAPK/ERK pathway. MedComm. 2025;6(3): Article e70126. 40066226 10.1002/mco2.70126 PMC11892147

[35] Plebanek MP, Xue Y, Nguyen YV, DeVito NC, Wang X, Holtzhausen A, Beasley GM, Theivanthiran B, Hanks BA. A lactate-SREBP2 signaling axis drives tolerogenic dendritic cell maturation and promotes cancer progression. Sci Immunol. 2024;9(95): Article eadi4191. 38728412 10.1126/sciimmunol.adi4191 PMC11926670

[36] Pucino V, Certo M, Bulusu V, Cucchi D, Goldmann K, Pontarini E, Haas R, Smith J, Headland SE, Blighe K, et al. Lactate buildup at the site of chronic inflammation promotes disease by inducing CD4+ T cell metabolic rewiring. Cell Metab. 2019;30(6):1055–1074.e8. 31708446 10.1016/j.cmet.2019.10.004 PMC6899510

[37] Chaudhari P, Madaan A, Rivera JC, Charfi I, Habelrih T, Hou X, Nezhady M, Lodygensky G, Pineyro G, Muanza T, et al. Neuronal GPR81 regulates developmental brain angiogenesis and promotes brain recovery after a hypoxic ischemic insult. J Cereb Blood Flow Metab. 2022;42(7):1294–1308. 35107038 10.1177/0271678X221077499 PMC9207492

[38] Brown TP, Ganapathy V. Lactate/GPR81 signaling and proton motive force in cancer: Role in angiogenesis, immune escape, nutrition, and Warburg phenomenon. Pharmacol Ther. 2020;206: Article 107451. 31836453 10.1016/j.pharmthera.2019.107451

[39] Chen S, Zhou X, Li W, Yang X, Niu X, Hu Z, Li S, Chen G, Sui X, Liu J, et al. Development of a novel peptide targeting GPR81 to suppress adipocyte-mediated tumor progression. Biochem Pharmacol. 2023;217: Article 115800. 37696459 10.1016/j.bcp.2023.115800

[40] Vohra R, Sanz-Morello B, Tams ALM, Mouhammad ZA, Freude KK, Hannibal J, Aldana BI, Bergersen LH, Kolko M. Prevention of cell death by activation of hydroxycarboxylic acid receptor 1 (GPR81) in retinal explants. Cells. 2022;11(13):2098. 35805182 10.3390/cells11132098 PMC9265426

[41] Su J, Mao X, Wang L, Chen Z, Wang W, Zhao C, Li G, Guo W, Hu Y. Lactate/GPR81 recruits regulatory T cells by modulating CX3CL1 to promote immune resistance in a highly glycolytic gastric cancer. Onco Targets Ther. 2024;13(1):2320951. 10.1080/2162402X.2024.2320951 PMC10900271 38419759

[42] Brown TP, Bhattacharjee P, Ramachandran S, Sivaprakasam S, Ristic B, Sikder MOF, Ganapathy V. The lactate receptor GPR81 promotes breast cancer growth via a paracrine mechanism involving antigen-presenting cells in the tumor microenvironment. Oncogene. 2020;39(16):3292–3304. 32071396 10.1038/s41388-020-1216-5

[43] Roland CL, Arumugam T, Deng D, Liu SH, Philip B, Gomez S, Burns WR, Ramachandran V, Wang H, Cruz-Monserrate Z, et al. Cell surface lactate receptor GPR81 is crucial for cancer cell survival. Cancer Res. 2014;74(18):5301–5310. 24928781 10.1158/0008-5472.CAN-14-0319 PMC4167222

[44] Qu J, Sun Z, Peng C, Li D, Yan W, Xu Z, Hou Y, Shen S, Chen P, Wang T. Tropicalis promotes chemotherapy resistance in colon cancer through increasing lactate production to regulate the mismatch repair system. Int J Biol Sci. 2021;17(11):2756–2769. 34345205 10.7150/ijbs.59262 PMC8326116

[45] Wagner W, Kania KD, Blauz A, Ciszewski WM. The lactate receptor (HCAR1/GPR81) contributes to doxorubicin chemoresistance via ABCB1 transporter up-regulation in human cervical cancer HeLa cells. J Physiol Pharmacol. 2017;68(4):555–564. 29151072

[46] Lee YJ, Shin KJ, Park SA, Park KS, Park S, Heo K, Seo YK, Noh DY, Ryu SH, Suh PG. G-protein-coupled receptor 81 promotes a malignant phenotype in breast cancer through angiogenic factor secretion. Oncotarget. 2016;7(43):70898–70911. 27765922 10.18632/oncotarget.12286 PMC5342597

[47] Lin Y, Jiang Y, Xian H, Cai X, Wang T. Expression and correlation of the Pi3k/Akt pathway and VEGF in oral submucous fibrosis. Cell Prolif. 2023;56(11): Article e13491. 37157945 10.1111/cpr.13491 PMC10623954

[48] Guo X, Wan P, Shen W, Sun M, Peng Z, Liao Y, Huang Y, Liu R. Fusobacterium periodonticum BCT protein targeting glucose metabolism to promote the epithelial-mesenchymal transition of esophageal cancer cells by lactic acid. J Transl Med. 2024;22(1):401. 38689341 10.1186/s12967-024-05157-z PMC11061911

[49] Feng G, Zhang L, Bao W, Ni J, Wang Y, Huang Y, Lyv J, Cao X, Chen T, You K, et al. Gentisic acid prevents colorectal cancer metastasis via blocking GPR81-mediated DEPDC5 degradation. Phytomedicine. 2024;129: Article 155615. 38615493 10.1016/j.phymed.2024.155615

[50] Zhi Y, Fan K, Liu S, Hu K, Zan X, Lin L, Yang Y, Gong X, Chen K, Tang L, et al. Deletion of GPR81 activates CREB/Smad7 pathway and alleviates liver fibrosis in mice. Mol Med. 2024;30(1):99. 38982366 10.1186/s10020-024-00867-y PMC11234765

[51] Ten Dijke P, Miyazono K, Heldin CH, Moustakas A. Special issue: TGF-β and epithelial-mesenchymal transition in cancer. Semin Cancer Biol. 2024;102-103:1–3. 38944133 10.1016/j.semcancer.2024.06.002

[52] Yang X, Lu Y, Hang J, Zhang J, Zhang T, Huo Y, Liu J, Lai S, Luo D, Wang L, et al. Lactate-modulated immunosuppression of myeloid-derived suppressor cells contributes to the radioresistance of pancreatic cancer. Cancer Immunol Res. 2020;8(11):1440–1451. 32917658 10.1158/2326-6066.CIR-20-0111

[53] Gyamfi J, Lee YH, Eom M, Choi J. Interleukin-6/STAT3 signalling regulates adipocyte induced epithelial-mesenchymal transition in breast cancer cells. Sci Rep. 2018;8(1):8859. 29891854 10.1038/s41598-018-27184-9 PMC5995871

[54] Hu Z, Sui Q, Jin X, Shan G, Huang Y, Yi Y, Zeng D, Zhao M, Zhan C, Wang Q, et al. IL6-STAT3-C/EBPβ-IL6 positive feedback loop in tumor-associated macrophages promotes the EMT and metastasis of lung adenocarcinoma. J Exp Clin Cancer Res. 2024;43(1):63. 38424624 10.1186/s13046-024-02989-x PMC10903044

[55] Jia C, Wang G, Wang T, Fu B, Zhang Y, Huang L, Deng Y, Chen G, Wu X, Chen J, et al. Cancer-associated fibroblasts induce epithelial-mesenchymal transition via the transglutaminase 2-dependent IL-6/IL6R/STAT3 axis in hepatocellular carcinoma. Int J Biol Sci. 2020;16(14):2542–2558. 32792856 10.7150/ijbs.45446 PMC7415430

[56] Liu X, Li S, Cui Q, Guo B, Ding W, Liu J, Quan L, Li X, Xie P, Jin L, et al. Activation of GPR81 by lactate drives tumour-induced cachexia. Nat Metab. 2024;6(4):708–723. 38499763 10.1038/s42255-024-01011-0 PMC11052724

[57] Moreno-Yruela C, Zhang D, Wei W, Bæk M, Liu W, Gao J, Danková D, Nielsen AL, Bolding JE, Yang L, et al. Class I histone deacetylases (HDAC1-3) are histone lysine delactylases. Sci Adv. 2022;8(3): Article eabi6696. 35044827 10.1126/sciadv.abi6696 PMC8769552

[58] Gaffney DO, Jennings EQ, Anderson CC, Marentette JO, Shi T, Schou Oxvig AM, Streeter MD, Johannsen M, Spiegel DA, Chapman E, et al. Non-enzymatic lysine lactoylation of glycolytic enzymes. Cell Chem Biol. 2020;27(2):206–213.e6. 31767537 10.1016/j.chembiol.2019.11.005 PMC7395678

[59] Li L, Li L, Li W, Chen T, Zou B, Zhao L, Wang H, Wang X, Xu L, Liu X, et al. TAp73-induced phosphofructokinase-1 transcription promotes the Warburg effect and enhances cell proliferation. Nat Commun. 2018;9(1):4683. 30409970 10.1038/s41467-018-07127-8 PMC6224601

[60] Lunt SY, Vander Heiden MG. Aerobic glycolysis: Meeting the metabolic requirements of cell proliferation. Annu Rev Cell Dev Biol. 2011;27:441–464. 21985671 10.1146/annurev-cellbio-092910-154237

[61] He J, Chai X, Zhang Q, Wang Y, Wang Y, Yang X, Wu J, Feng B, Sun J, Rui W, et al. The lactate receptor HCAR1 drives the recruitment of immunosuppressive PMN-MDSCs in colorectal cancer. Nat Immunol. 2025;26(3):391–403. 39905201 10.1038/s41590-024-02068-5

[62] Feng J, Yang H, Zhang Y, Wei H, Zhu Z, Zhu B, Yang M, Cao W, Wang L, Wu Z. Tumor cell-derived lactate induces TAZ-dependent upregulation of PD-L1 through GPR81 in human lung cancer cells. Oncogene. 2017;36(42):5829–5839. 28604752 10.1038/onc.2017.188

[63] Estrella V, Chen T, Lloyd M, Wojtkowiak J, Cornnell HH, Ibrahim-Hashim A, Bailey K, Balagurunathan Y, Rothberg JM, Sloane BF, et al. Acidity generated by the tumor microenvironment drives local invasion. Cancer Res. 2013;73(5):1524–1535. 23288510 10.1158/0008-5472.CAN-12-2796 PMC3594450

[64] Wang H, Cai J, Du S, Wei W, Shen X. LAMC2 modulates the acidity of microenvironments to promote invasion and migration of pancreatic cancer cells via regulating AKT-dependent NHE1 activity. Exp Cell Res. 2020;391(1): Article 111984. 32246993 10.1016/j.yexcr.2020.111984

[65] Lee S, Axelsen TV, Jessen N, Pedersen SF, Vahl P, Boedtkjer E. Na+,HCO3--cotransporter NBCn1 (Slc4a7) accelerates ErbB2-induced breast cancer development and tumor growth in mice. Oncogene. 2018;37(41):5569–5584. 29907770 10.1038/s41388-018-0353-6

[66] Xie R, Wang H, Jin H, Wen G, Tuo B, Xu J. NHE1 is upregulated in gastric cancer and regulates gastric cancer cell proliferation, migration and invasion. Oncol Rep. 2017;37(3):1451–1460. 28098891 10.3892/or.2017.5386

[67] Sun Z, Luan S, Yao Y, Qin T, Xu X, Shen Z, Yao R, Yue L. NHE1 mediates 5-Fu resistance in gastric cancer via STAT3 signaling pathway. Onco Targets Ther. 2020;24(13):8521–8532. 10.2147/OTT.S256274 PMC7457598 32904684

[68] Mo L, Xu L, Jia M, Su B, Hu Y, Hu Z, Li H, Zhao C, Zhao Z, Li J. Shikonin suppresses the epithelial-to-mesenchymal transition by downregulating NHE1 in bladder cancer cells. J Cancer. 2021;12(22):6814–6824. 34659570 10.7150/jca.63429 PMC8518005

[69] Lauritzen G, Stock CM, Lemaire J, Lund SF, Jensen MF, Damsgaard B, Petersen KS, Wiwel M, Rønnov-Jessen L, Schwab A, et al. The Na^+^/H^+^ exchanger NHE1, but not the Na^+^, HCO$\bar{3}$ cotransporter NBCn1, regulates motility of MCF7 breast cancer cells expressing constitutively active ErbB2. Cancer Lett. 2012;317(2):172–183. 22120673 10.1016/j.canlet.2011.11.023

[70] Flinck M, Kramer SH, Schnipper J, Andersen AP, Pedersen SF. The acid-base transport proteins NHE1 and NBCn1 regulate cell cycle progression in human breast cancer cells. Cell Cycle. 2018;17(9):1056–1067. 29895196 10.1080/15384101.2018.1464850 PMC6110587

[71] Niu D, Luo T, Wang H, Xia Y, Xie Z. Lactic acid in tumor invasion. Clin Chim Acta. 2021;522:61–69. 34400170 10.1016/j.cca.2021.08.011

[72] Vinay DS, Ryan EP, Pawelec G, Talib WH, Stagg J, Elkord E, Lichtor T, Decker WK, Whelan RL, Kumara HMCS, et al. Immune evasion in cancer: Mechanistic basis and therapeutic strategies. Semin Cancer Biol. 2015;35(Suppl):S185–S198. 25818339 10.1016/j.semcancer.2015.03.004

[73] Brand A, Singer K, Koehl GE, Kolitzus M, Schoenhammer G, Thiel A, Matos C, Bruss C, Klobuch S, Peter K, et al. LDHA-associated lactic acid production blunts tumor immunosurveillance by T and NK cells. Cell Metab. 2016;24(5):657–671. 27641098 10.1016/j.cmet.2016.08.011

[74] Fischer K, Hoffmann P, Voelkl S, Meidenbauer N, Ammer J, Edinger M, Gottfried E, Schwarz S, Rothe G, Hoves S, et al. Inhibitory effect of tumor cell-derived lactic acid on human T cells. Blood. 2007;109(9):3812–3819. 17255361 10.1182/blood-2006-07-035972

[75] Colegio OR, Chu NQ, Szabo AL, Chu T, Rhebergen AM, Jairam V, Cyrus N, Brokowski CE, Eisenbarth SC, Phillips GM, et al. Functional polarization of tumour-associated macrophages by tumour-derived lactic acid. Nature. 2014;513(7519):559–563. 25043024 10.1038/nature13490 PMC4301845

[76] Xiong J, He J, Zhu J, Pan J, Liao W, Ye H, Wang H, Song Y, Du Y, Cui B, et al. Lactylation-driven METTL3-mediated RNA m6A modification promotes immunosuppression of tumor-infiltrating myeloid cells. Mol Cell. 2022;82(9):1660–1677.e10. 35320754 10.1016/j.molcel.2022.02.033

[77] Chaudagar K, Hieromnimon HM, Khurana R, Labadie B, Hirz T, Mei S, Hasan R, Shafran J, Kelley A, Apostolov E, et al. Reversal of lactate and PD-1-mediated macrophage immunosuppression controls growth of PTEN/p53-deficient prostate cancer. Clin Cancer Res. 2023;29(10):1952–1968. 36862086 10.1158/1078-0432.CCR-22-3350 PMC10192075

[78] Forner A, Reig M, Bruix J. Hepatocellular carcinoma. Lancet. 2018;391(10127):1301–1314. 29307467 10.1016/S0140-6736(18)30010-2

[79] Alawyia B, Constantinou C. Hepatocellular carcinoma: A narrative review on current knowledge and future prospects. Curr Treat Options in Oncol. 2023;24(7):711–724. 10.1007/s11864-023-01098-9 37103744

[80] Ziki RA, Colnot S. Glutamine metabolism, a double agent combating or fuelling hepatocellular carcinoma. JHEP Rep. 2024;6(5): Article 101077. 38699532 10.1016/j.jhepr.2024.101077 PMC11063524

[81] Ma H, Yang L, Liang Y, Liu F, Hu J, Zhang R, Li Y, Yuan L, Feng F. B. Thetaiotaomicron-derived acetic acid modulate immune microenvironment and tumor growth in hepatocellular carcinoma. Gut Microbes. 2024;16(1): Article 2297846. 38270111 10.1080/19490976.2023.2297846 PMC10813637

[82] Su Y, Li Z, Li Q, Guo X, Zhang H, Li Y, Meng Z, Huang S, Hu Z. Oncofetal TRIM71 drives liver cancer carcinogenesis through remodeling CEBPA-mediated serine/glycine metabolism. Theranostics. 2024;14(13):4948–4966. 39267787 10.7150/thno.99633 PMC11388079

[83] Ladd AD, Duarte S, Sahin I, Zarrinpar A. Mechanisms of drug resistance in HCC. Hepatology. 2024;79(4):926–940. 36680397 10.1097/HEP.0000000000000237

[84] Yang Z, Yan C, Ma J, Peng P, Ren X, Cai S, Shen X, Wu Y, Zhang S, Wang X, et al. Lactylome analysis suggests lactylation-dependent mechanisms of metabolic adaptation in hepatocellular carcinoma. Nat Metab. 2023;5(1):61–79. 36593272 10.1038/s42255-022-00710-w

[85] Pan L, Feng F, Wu J, Fan S, Han J, Wang S, Yang L, Liu W, Wang C, Xu K. Demethylzeylasteral targets lactate by inhibiting histone lactylation to suppress the tumorigenicity of liver cancer stem cells. Pharmacol Res. 2022;181: Article 106270. 35605812 10.1016/j.phrs.2022.106270

[86] Sun JH, Luo Q, Liu LL, Song GB. Liver cancer stem cell markers: Progression and therapeutic implications. World J Gastroenterol. 2016;22(13):3547–3557. 27053846 10.3748/wjg.v22.i13.3547 PMC4814640

[87] Wong CC, Yu J. Gut microbiota in colorectal cancer development and therapy. Nat Rev Clin Oncol. 2023;20(7):429–452. 37169888 10.1038/s41571-023-00766-x

[88] Cheng X, Wang H, Wang Z, Zhu B, Long H. Tumor-associated myeloid cells in cancer immunotherapy. J Hematol Oncol. 2023;16(1):71. 37415162 10.1186/s13045-023-01473-x PMC10324139

[89] Zhu W, Si Y, Xu J, Lin Y, Wang JZ, Cao M, Sun S, Ding Q, Zhu L, Wei JF. Methyltransferase like 3 promotes colorectal cancer proliferation by stabilizing CCNE1 mRNA in an m6A-dependent manner. J Cell Mol Med. 2020;24(6):3521–3533. 32039568 10.1111/jcmm.15042 PMC7131945

[90] Gu J, Xu X, Li X, Yue L, Zhu X, Chen Q, Gao J, Takashi M, Zhao W, Zhao B, et al. Tumor-resident microbiota contributes to colorectal cancer liver metastasis by lactylation and immune modulation. Oncogene. 2024;43(31):2389–2404. 38890429 10.1038/s41388-024-03080-7 PMC11281901

[91] Sun Q, Wu J, Zhu G, Li T, Zhu X, Ni B, Xu B, Ma X, Li J. Lactate-related metabolic reprogramming and immune regulation in colorectal cancer. Front Endocrinol. 2023;26(13):1089918. 10.3389/fendo.2022.1089918 PMC9909490 36778600

[92] Wang X, Feng J, Xue Y, Guan Z, Zhang D, Liu Z, Gong Z, Wang Q, Huang J, Tang C, et al. Structural basis of N^6^-adenosine methylation by the METTL3-METTL14 complex. Nature. 2016;534(7608):575–578. 27281194 10.1038/nature18298

[93] Miao Z, Zhao X, Liu X. Hypoxia induced β-catenin lactylation promotes the cell proliferation and stemness of colorectal cancer through the wnt signaling pathway. Exp Cell Res. 2023;422(1): Article 113439. 36464122 10.1016/j.yexcr.2022.113439

[94] Herbst RS, Morgensztern D, Boshoff C. The biology and management of non-small cell lung cancer. Nature. 2018;553(7689):446–454. 29364287 10.1038/nature25183

[95] Jiang J, Huang D, Jiang Y, Hou J, Tian M, Li J, Sun L, Zhang Y, Zhang T, Li Z, et al. Lactate modulates cellular metabolism through histone lactylation-mediated gene expression in non-small cell lung cancer. Front Oncol. 2021;2(11): Article 647559. 10.3389/fonc.2021.647559 PMC8208031 34150616

[96] Dai J, Lu X, Zhang C, Qu T, Li W, Su J, Guo R, Yin D, Wu P, Han L, et al. NNMT promotes acquired EGFR-TKI resistance by forming EGR1 and lactate-mediated double positive feedback loops in non-small cell lung cancer. Mol Cancer. 2025;24(1):79. 40089784 10.1186/s12943-025-02285-y PMC11909984

[97] Bobos M. Histopathologic classification and prognostic factors of melanoma: A 2021 update. Ital J Dermatol Venerol. 2021;156(3):300–321. 33982546 10.23736/S2784-8671.21.06958-3

[98] Tasdogan A, Faubert B, Ramesh V, Ubellacker JM, Shen B, Solmonson A, Murphy MM, Gu Z, Gu W, Martin M, et al. Metabolic heterogeneity confers differences in melanoma metastatic potential. Nature. 2020;577(7788):115–120. 31853067 10.1038/s41586-019-1847-2 PMC6930341

[99] Gopal YN, Rizos H, Chen G, Deng W, Frederick DT, Cooper ZA, Scolyer RA, Pupo G, Komurov K, Sehgal V, et al. Inhibition of mTORC1/2 overcomes resistance to MAPK pathway inhibitors mediated by PGC1α and oxidative phosphorylation in melanoma. Cancer Res. 2014;74(23):7037–7047. 25297634 10.1158/0008-5472.CAN-14-1392 PMC4347853

[100] Longhitano L, Giallongo S, Orlando L, Broggi G, Longo A, Russo A, Caltabiano R, Giallongo C, Barbagallo I, Di Rosa M, et al. Lactate rewrites the metabolic reprogramming of uveal melanoma cells and induces quiescence phenotype. Int J Mol Sci. 2022;24(1):24. 36613471 10.3390/ijms24010024 PMC9820521

[101] Yu J, Chai P, Xie M, Ge S, Ruan J, Fan X, Jia R. Histone lactylation drives oncogenesis by facilitating m6A reader protein YTHDF2 expression in ocular melanoma. Genome Biol. 2021;22(1):85. 33726814 10.1186/s13059-021-02308-z PMC7962360

[102] Bahadoram S, Davoodi M, Hassanzadeh S, Bahadoram M, Barahman M, Mafakher L. Renal cell carcinoma: An overview of the epidemiology, diagnosis, and treatment. G Ital Nefrol. 2022;39(3):2022. 35819037

[103] Escudier B, Porta C, Schmidinger M, Rioux-Leclercq N, Bex A, Khoo V, Grünwald V, Gillessen S, Horwich A, ESMO Guidelines Committee. Renal cell carcinoma: ESMO Clinical Practice Guidelines for diagnosis, treatment and follow-up. Ann Oncol. 2019;30(5):706–720. 30788497 10.1093/annonc/mdz056

[104] Kong W, He J, Zhou Q, Zhou X, Wei X, Yang Y, Mei Y, Wang S, Zhang X, Yao B, et al. Histone lactylation-related genes correlate with the molecular patterns and functions of cancer-associated fibroblasts and have significant clinical implications in clear cell renal cell carcinoma. Heliyon. 2024;10(13): Article e33554. 39035489 10.1016/j.heliyon.2024.e33554 PMC11259888

[105] Yang L, Wang X, Liu J, Liu X, Li S, Zheng F, Dong Q, Xu S, Xiong J, Fu B. Prognostic and tumor microenvironmental feature of clear cell renal cell carcinoma revealed by m6A and lactylation modification-related genes. Front Immunol. 2023;11(14):1225023. 10.3389/fimmu.2023.1225023 PMC10450969 37638005

[106] Yang J, Luo L, Zhao C, Li X, Wang Z, Zeng Z, Yang X, Zheng X, Jie H, Kang L, et al. A positive feedback loop between inactive VHL-triggered histone lactylation and PDGFRβ signaling drives clear cell renal cell carcinoma progression. Int J Biol Sci. 2022;18(8):3470–3483. 35637958 10.7150/ijbs.73398 PMC9134910

[107] Zhang C, Wang QT, Liu H, Zhang ZZ, Huang WL. Advancement and prospects of tumor gene therapy. Chin J Cancer. 2011;30(3):182–188. 21352695 10.5732/cjc.010.10074 PMC4013314

[108] Mahmood-ur-Rahman, Ali I, Husnain T, Riazuddin S. RNA interference: The story of gene silencing in plants and humans. Biotechnol Adv. 2008;26(3):202–209. 18221853 10.1016/j.biotechadv.2007.12.002

[109] Yu H, Yin Y, Yi Y, Cheng Z, Kuang W, Li R, Zhong H, Cui Y, Yuan L, Gong F, et al. Targeting lactate dehydrogenase A (LDHA) exerts antileukemic effects on T-cell acute lymphoblastic leukemia. Cancer Commun. 2020;40(10):501–517. 10.1002/cac2.12080 PMC7571401 32820611

[110] Spencer TL, Lehninger AL. L-lactate transport in Ehrlich ascites-tumour cells. Biochem J. 1976;154(2):405–414. 7237 10.1042/bj1540405 PMC1172721

[111] Alobaidi B, Hashimi SM, Alqosaibi AI, AlQurashi N, Alhazmi S. Targeting the monocarboxylate transporter MCT2 and lactate dehydrogenase A LDHA in cancer cells with FX-11 and AR-C155858 inhibitors. Eur Rev Med Pharmacol Sci. 2023;27(14):6605–6617. 37522672 10.26355/eurrev_202307_33131

[112] Jones RS, Morris ME. Monocarboxylate transporters: Therapeutic targets and prognostic factors in disease. Clin Pharmacol Ther. 2016;100(5):454–463. 27351344 10.1002/cpt.418 PMC5533588

[113] Afonso J, Santos LL, Miranda-Gonçalves V, Morais A, Amaro T, Longatto-Filho A, Baltazar F. CD147 and MCT1-potential partners in bladder cancer aggressiveness and cisplatin resistance. Mol Carcinog. 2015;54(11):1451–1466. 25263481 10.1002/mc.22222

[114] Miranda-Gonçalves V, Honavar M, Pinheiro C, Martinho O, Pires MM, Pinheiro C, Cordeiro M, Bebiano G, Costa P, Palmeirim I, et al. Monocarboxylate transporters (MCTs) in gliomas: Expression and exploitation as therapeutic targets. Neuro-Oncology. 2013;15(2):172–188. 23258846 10.1093/neuonc/nos298 PMC3548586

[115] Kennedy KM, Dewhirst MW. Tumor metabolism of lactate: The influence and therapeutic potential for MCT and CD147 regulation. Future Oncol. 2010;6(1):127–148. 20021214 10.2217/fon.09.145 PMC2819205

[116] Kobayashi M, Narumi K, Furugen A, Iseki K. Transport function, regulation, and biology of human monocarboxylate transporter 1 (hMCT1) and 4 (hMCT4). Pharmacol Ther. 2021;226: Article 107862. 33894276 10.1016/j.pharmthera.2021.107862

[117] Koukourakis MI, Giatromanolaki A, Winter S, Leek R, Sivridis E, Harris AL. Lactate dehydrogenase 5 expression in squamous cell head and neck cancer relates to prognosis following radical or postoperative radiotherapy. Oncology. 2009;77(5):285–292. 19923867 10.1159/000259260

[118] Le A, Cooper CR, Gouw AM, Dinavahi R, Maitra A, Deck LM, Royer RE, Vander Jagt DL, Semenza GL, Dang CV. Inhibition of lactate dehydrogenase a induces oxidative stress and inhibits tumor progression. Proc Natl Acad Sci USA. 2010;107(5):2037–2042. 20133848 10.1073/pnas.0914433107 PMC2836706

[119] Billiard J, Dennison JB, Briand J, Annan RS, Chai D, Colón M, Dodson CS, Gilbert SA, Greshock J, Jing J, et al. Quinoline 3-sulfonamides inhibit lactate dehydrogenase A and reverse aerobic glycolysis in cancer cells. Cancer Metab. 2013;1(1):19. 24280423 10.1186/2049-3002-1-19 PMC4178217

[120] Allison SJ, Knight JR, Granchi C, Rani R, Minutolo F, Milner J, Phillips RM. Identification of LDH-A as a therapeutic target for cancer cell killing via (i) p53/NAD(H)-dependent and (ii) p53-independent pathways. Oncogenesis. 2014;3(5): Article e102. 24819061 10.1038/oncsis.2014.16 PMC4035693

[121] Granchi C, Roy S, Giacomelli C, Macchia M, Tuccinardi T, Martinelli A, Lanza M, Betti L, Giannaccini G, Lucacchini A, et al. Discovery of N-hydroxyindole-based inhibitors of human lactate dehydrogenase isoform A (LDH-A) as starvation agents against cancer cells. J Med Chem. 2011;54(6):1599–1612. 21332213 10.1021/jm101007q

[122] Rani R, Kumar V. Recent update on human lactate dehydrogenase enzyme 5 (hLDH5) inhibitors: A promising approach for cancer chemotherapy. J Med Chem. 2016;59(2):487–496. 26340601 10.1021/acs.jmedchem.5b00168

[123] Shelley MD, Hartley L, Fish RG, Groundwater P, Morgan JJ, Mort D, Mason M, Evans A. Stereo-specific cytotoxic effects of gossypol enantiomers and gossypolone in tumour cell lines. Cancer Lett. 1999;135(2):171–180. 10096426 10.1016/s0304-3835(98)00302-4

[124] Van Poznak C, Seidman AD, Reidenberg MM, Moasser MM, Sklarin N, Van Zee K, Borgen P, Gollub M, Bacotti D, Yao TJ, et al. Oral gossypol in the treatment of patients with refractory metastatic breast cancer: A phase I/II clinical trial. Breast Cancer Res Treat. 2001;66(3):239–248. 11510695 10.1023/a:1010686204736

[125] Rellinger EJ, Craig BT, Alvarez AL, Dusek HL, Kim KW, Qiao J, Chung DH. FX11 inhibits aerobic glycolysis and growth of neuroblastoma cells. Surgery. 2017;161(3):747–752. 27919448 10.1016/j.surg.2016.09.009 PMC5369647

[126] Zhai X, Yang Y, Wan J, Zhu R, Wu Y. Inhibition of LDH-A by oxamate induces G2/M arrest, apoptosis and increases radiosensitivity in nasopharyngeal carcinoma cells. Oncol Rep. 2013;30(6):2983–2991. 24064966 10.3892/or.2013.2735

[127] Zhao Z, Han F, Yang S, Wu J, Zhan W. Oxamate-mediated inhibition of lactate dehydrogenase induces protective autophagy in gastric cancer cells: Involvement of the Akt-mTOR signaling pathway. Cancer Lett. 2015;358(1):17–26. 25524555 10.1016/j.canlet.2014.11.046

[128] Friberg A, Rehwinkel H, Nguyen D, Pütter V, Quanz M, Weiske J, Eberspächer U, Heisler I, Langer G. Structural evidence for isoform-selective allosteric inhibition of lactate dehydrogenase A. ACS Omega. 2020;5(22):13034–13041. 32548488 10.1021/acsomega.0c00715 PMC7288559

[129] Zaimy MA, Saffarzadeh N, Mohammadi A, Pourghadamyari H, Izadi P, Sarli A, Moghaddam LK, Paschepari SR, Azizi H, Torkamandi S, et al. New methods in the diagnosis of cancer and gene therapy of cancer based on nanoparticles. Cancer Gene Ther. 2017;24(6):233–243. 28574057 10.1038/cgt.2017.16

[130] Zamboni WC. Liposomal, nanoparticle, and conjugated formulations of anticancer agents. Clin Cancer Res. 2005;11(23):8230–8234. 16322279 10.1158/1078-0432.CCR-05-1895

[131] Zhang XP, Sun JG, Yao J, Shan K, Liu BH, Yao MD, Ge HM, Jiang Q, Zhao C, Yan B. Effect of nanoencapsulation using poly (lactide-co-glycolide) (PLGA) on anti-angiogenic activity of bevacizumab for ocular angiogenesis therapy. Biomed Pharmacother. 2018;107:1056–1063. 30257317 10.1016/j.biopha.2018.08.092

[132] Park K. Nanotechnology: What it can do for drug delivery. J Control Release. 2007;120(1-2):1–3. 17532520 10.1016/j.jconrel.2007.05.003 PMC1949907

[133] Parveen S, Sahoo SK. Long circulating chitosan/PEG blended PLGA nanoparticle for tumor drug delivery. Eur J Pharmacol. 2011;670(2-3):372–383. 21951969 10.1016/j.ejphar.2011.09.023

[134] Sadat Tabatabaei Mirakabad F, Nejati-Koshki K, Akbarzadeh A, Yamchi MR, Milani M, Zarghami N, Zeighamian V, Rahimzadeh A, Alimohammadi S, Hanifehpour Y, et al. PLGA-based nanoparticles as cancer drug delivery systems. Asian Pac J Cancer Prev. 2014;15(2):517–535. 24568455 10.7314/apjcp.2014.15.2.517

[135] Caputo TM, Cusano AM, Principe S, Cicatiello P, Celetti G, Aliberti A, Micco A, Ruvo M, Tagliamonte M, Ragone C, et al. Sorafenib-loaded PLGA carriers for enhanced drug delivery and cellular uptake in liver cancer cells. Int J Nanomedicine. 2023;26(18):4121–4142. 10.2147/IJN.S415968 PMC10387258 37525693

[136] Lin W, Li C, Xu N, Watanabe M, Xue R, Xu A, Araki M, Sun R, Liu C, Nasu Y, et al. Dual-functional PLGA nanoparticles co-loaded with indocyanine green and resiquimod for prostate cancer treatment. Int J Nanomedicine. 2021;12(16):2775–2787. 10.2147/IJN.S301552 PMC8052122 33880023

[137] Liu J, Huang H, Zhang X, Shen Y, Jiang D, Hu S, Li S, Yan Z, Hu W, Luo J, et al. Unveiling the cuproptosis in colitis and colitis-related carcinogenesis: A multifaceted player and immune moderator. Research. 2025;14(8):0698. 10.34133/research.0698 PMC12076167 40370501

[138] Nwazojie CC, Obayemi JD, Salifu AA, Borbor-Sawyer SM, Uzonwanne VO, Onyekanne CE, Akpan UM, Onwudiwe KC, Oparah JC, Odusanya OS, et al. Targeted drug-loaded PLGA-PCL microspheres for specific and localized treatment of triple negative breast cancer. J Mater Sci Mater Med. 2023;34(8):41. 37530973 10.1007/s10856-023-06738-y PMC10397127

[139] Zhang HT, Peng R, Chen S, Shen A, Zhao L, Tang W, Wang XH, Li ZY, Zha ZG, Yi M, et al. Versatile nano-PROTAC-induced epigenetic reader degradation for efficient lung cancer therapy. Adv Sci. 2022;9(29): Article e2202039. 10.1002/advs.202202039 PMC9561860 35988145

[140] Hua S, Jeong HN, Dimapasoc LM, Kang I, Han C, Choi JS, Lebrilla CB, An HJ. Isomer-specific LC/MS and LC/MS/MS profiling of the mouse serum N-glycome revealing a number of novel sialylated N-glycans. Anal Chem. 2013;85(9):4636–4643. 23534819 10.1021/ac400195h PMC5636693

[141] Hua S, Lebrilla C, An HJ. Application of nano-LC-based glycomics towards biomarker discovery. Bioanalysis. 2011;3(22):2573–2585. 22122604 10.4155/bio.11.263

[142] Campbell MP, Royle L, Radcliffe CM, Dwek RA, Rudd PM. GlycoBase and autoGU: Tools for HPLC-based glycan analysis. Bioinformatics. 2008;24(9):1214–1216. 18344517 10.1093/bioinformatics/btn090

[143] Aldredge D, An HJ, Tang N, Waddell K, Lebrilla CB. Annotation of a serum N-glycan library for rapid identification of structures. J Proteome Res. 2012;11(3):1958–1968. 22320385 10.1021/pr2011439 PMC3292799

[144] Wu S, Salcedo J, Tang N, Waddell K, Grimm R, German JB, Lebrilla CB. Employment of tandem mass spectrometry for the accurate and specific identification of oligosaccharide structures. Anal Chem. 2012;84(17):7456–7462. 22867103 10.1021/ac301398h PMC3555499

[145] An HJ, Gip P, Kim J, Wu S, Park KW, CT MV, Schaffer DV, Bertozzi CR, Lebrilla CB. Extensive determination of glycan heterogeneity reveals an unusual abundance of high mannose glycans in enriched plasma membranes of human embryonic stem cells. Mol Cell Proteomics. 2012;11(4): Article M111.010660. 22147732 10.1074/mcp.M111.010660 PMC3322563

[146] Totten SM, Zivkovic AM, Wu S, Ngyuen U, Freeman SL, Ruhaak LR, Darboe MK, German JB, Prentice AM, Lebrilla CB. Comprehensive profiles of human milk oligosaccharides yield highly sensitive and specific markers for determining secretor status in lactating mothers. J Proteome Res. 2012;11(12):6124–6133. 23140396 10.1021/pr300769g

[147] Tao N, Wu S, Kim J, An HJ, Hinde K, Power ML, Gagneux P, German JB, Lebrilla CB. Evolutionary glycomics: Characterization of milk oligosaccharides in primates. J Proteome Res. 2011;10(4):1548–1557. 21214271 10.1021/pr1009367 PMC3070053

[148] Hua S, An HJ, Ozcan S, Ro GS, Soares S, DeVere-White R, Lebrilla CB. Comprehensive native glycan profiling with isomer separation and quantitation for the discovery of cancer biomarkers. Analyst. 2011;136(18):3663–3671. 21776491 10.1039/c1an15093f PMC3331797

[149] Hua S, Williams CC, Dimapasoc LM, Ro GS, Ozcan S, Miyamoto S, Lebrilla CB, An HJ, Leiserowitz GS. Isomer-specific chromatographic profiling yields highly sensitive and specific potential N-glycan biomarkers for epithelial ovarian cancer. J Chromatogr A. 2013;1279:58–67. 23380366 10.1016/j.chroma.2012.12.079 PMC5628020

[150] Oh MJ, Hua S, Kim BJ, Jeong HN, Jeong SH, Grimm R, Yoo JS, An HJ. Analytical platform for glycomic characterization of recombinant erythropoietin biotherapeutics and biosimilars by MS. Bioanalysis. 2013;5(5):545–559. 23425271 10.4155/bio.12.327

[151] Song Y, Liu X, Stielow JB, de Hoog S, Li R. Post-translational changes in *Phialophora verrucosa via* lysine lactylation during prolonged presence in a patient with a *CARD9*-related immune disorder. Front Immunol. 2022;8(13): Article 966457. 10.3389/fimmu.2022.966457 PMC9395174 36003392

[152] Xiao Y, Yu D. Tumor microenvironment as a therapeutic target in cancer. Pharmacol Ther. 2021;221: Article 107753. 33259885 10.1016/j.pharmthera.2020.107753 PMC8084948

[153] Hanahan D, Weinberg RA. Hallmarks of cancer: The next generation. Cell. 2011;144(5):646–674. 21376230 10.1016/j.cell.2011.02.013

[154] Hanahan D, Coussens LM. Accessories to the crime: Functions of cells recruited to the tumor microenvironment. Cancer Cell. 2012;21(3):309–322. 22439926 10.1016/j.ccr.2012.02.022

[155] Lyssiotis CA, Kimmelman AC. Metabolic interactions in the tumor microenvironment. Trends Cell Biol. 2017;27(11):863–875. 28734735 10.1016/j.tcb.2017.06.003 PMC5814137

[156] Hyeon DY, Nam D, Shin HJ, Jeong J, Jung E, Cho SY, Shin DH, Ku JL, Baek HJ, Yoo CW, et al. Proteogenomic characterization of molecular and cellular targets for treatment-resistant subtypes in locally advanced cervical cancers. Mol Cancer. 2025;24(1):77. 40087745 10.1186/s12943-025-02256-3 PMC11908047

[157] Sharma P, Hu-Lieskovan S, Wargo JA, Ribas A. Primary, adaptive, and acquired resistance to cancer immunotherapy. Cell. 2017;168(4):707–723. 28187290 10.1016/j.cell.2017.01.017 PMC5391692

[158] Li D, Wang Z, Yu Q, Wang J, Wu R, Tuo Z, Yoo KH, Wusiman D, Ye L, Guo Y, et al. Tracing the evolution of sex hormones and receptor-mediated immune microenvironmental differences in prostate and bladder cancers: From embryonic development to disease. Adv Sci. 2025;12(13): Article e2407715. 10.1002/advs.202407715 PMC11967776 40007149

[159] Yang J, Yu X, Xiao M, Xu H, Tan Z, Lei Y, Guo Y, Wang W, Xu J, Shi S, et al. Histone lactylation-driven feedback loop modulates cholesterol-linked immunosuppression in pancreatic cancer. Gut. 2025. 10.1136/gutjnl-2024-334361 PMC12573332 40467104

[160] Liu L, Li Y, Li B. Interactions between cancer cells and tumor-associated macrophages in tumor microenvironment. Biochim Biophys Acta Rev Cancer. 2025;1880(3): Article 189344. 40345263 10.1016/j.bbcan.2025.189344

[161] Cai J, Zhang P, Cai Y, Zhu G, Chen S, Song L, Du J, Wang B, Dai W, Zhou J, et al. Lactylation-driven NUPR1 promotes immunosuppression of tumor-infiltrating macrophages in hepatocellular carcinoma. Adv Sci. 2025;12(20): Article e2413095. 10.1002/advs.202413095 PMC12120759 40305758

[162] Chen Q, Yuan H, Bronze MS, Li M. Targeting lactylation and the STAT3/CCL2 axis to overcome immunotherapy resistance in pancreatic ductal adenocarcinoma. J Clin Invest. 2025;135(7): Article e191422. 40166931 10.1172/JCI191422 PMC11957682

[163] Raychaudhuri D, Singh P, Chakraborty B, Hennessey M, Tannir AJ, Byregowda S, Natarajan SM, Trujillo-Ocampo A, Im JS, Goswami S. Histone lactylation drives CD8^+^ T cell metabolism and function. Nat Immunol. 2024;25(11):2140–2151. 39375549 10.1038/s41590-024-01985-9 PMC13211864

[164] Xue Q, Peng W, Zhang S, Wei X, Ye L, Wang Z, Xiang X, Liu Y, Wang H, Zhou Q. Lactylation-driven TNFR2 expression in regulatory T cells promotes the progression of malignant pleural effusion. J Immunother Cancer. 2024;12(12): Article e010040. 39721754 10.1136/jitc-2024-010040 PMC11683941

[165] Lv B, Wang Y, Ma D, Cheng W, Liu J, Yong T, Chen H, Wang C. Immunotherapy: Reshape the tumor immune microenvironment. Front Immunol. 2022;6(13): Article 844142. 10.3389/fimmu.2022.844142 PMC9299092 35874717

[166] St Paul M, Ohashi PS. The roles of CD8^+^ T cell subsets in antitumor immunity. Trends Cell Biol. 2020;30(9):695–704. 32624246 10.1016/j.tcb.2020.06.003

[167] Cao T, Zhang W, Wang Q, Wang C, Ma W, Zhang C, Ge M, Tian M, Yu J, Jiao A, et al. Cancer SLC6A6-mediated taurine uptake transactivates immune checkpoint genes and induces exhaustion in CD8^+^ T cells. Cell. 2024;187(9):2288–2304.e27. 38565142 10.1016/j.cell.2024.03.011

[168] Wherry EJ. T cell exhaustion. Nat Immunol. 2011;12(6):492–499. 21739672 10.1038/ni.2035

[169] Ma Z, Yang J, Jia W, Li L, Li Y, Hu J, Luo W, Li R, Ye D, Lan P. Histone lactylation-driven B7-H3 expression promotes tumor immune evasion. Theranostics. 2025;15(6):2338–2359. 39990209 10.7150/thno.105947 PMC11840737

[170] Zhang C, Zhou L, Zhang M, Du Y, Li C, Ren H, Zheng L. H3K18 Lactylation potentiates immune escape of non-small cell lung cancer. Cancer Res. 2024;84(21):3589–3601. 39137401 10.1158/0008-5472.CAN-23-3513

[171] Huang ZW, Zhang XN, Zhang L, Liu LL, Zhang JW, Sun YX, Xu JQ, Liu Q, Long ZJ. STAT5 promotes PD-L1 expression by facilitating histone lactylation to drive immunosuppression in acute myeloid leukemia. Signal Transduct Target Ther. 2023;8(1):391. 37777506 10.1038/s41392-023-01605-2 PMC10542808

[172] Tong H, Jiang Z, Song L, Tan K, Yin X, He C, Huang J, Li X, Jing X, Yun H, et al. Dual impacts of serine/glycine-free diet in enhancing antitumor immunity and promoting evasion via PD-L1 lactylation. Cell Metab. 2024;36(12):2493–2510.e9. 39577415 10.1016/j.cmet.2024.10.019

[173] Wang R, Li C, Cheng Z, Li M, Shi J, Zhang Z, Jin S, Ma H. H3K9 lactylation in malignant cells facilitates CD8^+^ T cell dysfunction and poor immunotherapy response. Cell Rep. 2024;43(9): Article 114686. 39216002 10.1016/j.celrep.2024.114686

[174] Zhou C, Li W, Liang Z, Wu X, Cheng S, Peng J, Zeng K, Li W, Lan P, Yang X, et al. Mutant KRAS-activated circATXN7 fosters tumor immunoescape by sensitizing tumor-specific T cells to activation-induced cell death. Nat Commun. 2024;15(1):499. 38216551 10.1038/s41467-024-44779-1 PMC10786880

[175] Groth C, Maric J, Garcés Lázaro I, Hofman T, Zhang Z, Ni Y, Keller F, Seufert I, Hofmann M, Neumann-Haefelin C, et al. Hepatitis D infection induces IFN-β-mediated NK cell activation and TRAIL-dependent cytotoxicity. Front Immunol. 2023;14:1287367. 38143742 10.3389/fimmu.2023.1287367 PMC10739304

[176] Liu S, Galat V, Galat Y, Lee YKA, Wainwright D, Wu J. NK cell-based cancer immunotherapy: From basic biology to clinical development. J Hematol Oncol. 2021;14(1):7. 33407739 10.1186/s13045-020-01014-w PMC7788999

[177] Terrén I, Orrantia A, Vitallé J, Zenarruzabeitia O, Borrego F. NK cell metabolism and tumor microenvironment. Front Immunol. 2019;10:2278. 31616440 10.3389/fimmu.2019.02278 PMC6769035

[178] Poznanski SM, Singh K, Ritchie TM, Aguiar JA, Fan IY, Portillo AL, Rojas EA, Vahedi F, El-Sayes A, Xing S, et al. Metabolic flexibility determines human NK cell functional fate in the tumor microenvironment. Cell Metab. 2021;33(6):1205–1220.e5. 33852875 10.1016/j.cmet.2021.03.023

[179] Wu Z, Wu H, Dai Y, Wang Z, Han H, Shen Y, Zhang R, Wang X. A pan-cancer multi-omics analysis of lactylation genes associated with tumor microenvironment and cancer development. Heliyon. 2024;10(5): Article e27465. 38463768 10.1016/j.heliyon.2024.e27465 PMC10923869

[180] Wu Q, Li X, Long M, Xie X, Liu Q. Integrated analysis of histone lysine lactylation (Kla)-specific genes suggests that NR6A1, OSBP2 and UNC119B are novel therapeutic targets for hepatocellular carcinoma. Sci Rep. 2023;13(1):18642. 37903971 10.1038/s41598-023-46057-4 PMC10616101

[181] Wang ZH, Zhang P, Peng WB, Ye LL, Xiang X, Wei XS, Niu YR, Zhang SY, Xue QQ, Wang HL, et al. Altered phenotypic and metabolic characteristics of FOXP3^+^CD3^+^CD56^+^ natural killer T (NKT)-like cells in human malignant pleural effusion. Onco Targets Ther. 2022;12(1):2160558. 10.1080/2162402X.2022.2160558 PMC9788685 36567801

[182] Zhang Y, Cai K, Li C, Guo Q, Chen Q, He X, Liu L, Zhang Y, Lu Y, Chen X, et al. Macrophage-membrane-coated nanoparticles for tumor-targeted chemotherapy. Nano Lett. 2018;18(3):1908–1915. 29473753 10.1021/acs.nanolett.7b05263 PMC7470025

[183] Mehla K, Singh PK. Metabolic regulation of macrophage polarization in cancer. Trends Cancer. 2019;5(12):822–834. 31813459 10.1016/j.trecan.2019.10.007 PMC7187927

[184] Noe JT, Rendon BE, Geller AE, Conroy LR, Morrissey SM, Young LEA, Bruntz RC, Kim EJ, Wise-Mitchell A, de Souza B, et al. Lactate supports a metabolic-epigenetic link in macrophage polarization. Sci Adv. 2021;7(46): Article eabi8602. 34767443 10.1126/sciadv.abi8602 PMC8589316

[185] Chen P, Zuo H, Xiong H, Kolar MJ, Chu Q, Saghatelian A, Siegwart DJ, Wan Y. Gpr132 sensing of lactate mediates tumor-macrophage interplay to promote breast cancer metastasis. Proc Natl Acad Sci USA. 2017;114(3):580–585. 28049847 10.1073/pnas.1614035114 PMC5255630

[186] Wang L, Li S, Luo H, Lu Q, Yu S. PCSK9 promotes the progression and metastasis of colon cancer cells through regulation of EMT and PI3K/AKT signaling in tumor cells and phenotypic polarization of macrophages. J Exp Clin Cancer Res. 2022;41(1):303. 36242053 10.1186/s13046-022-02477-0 PMC9563506

[187] Chaudagar K, Hieromnimon HM, Kelley A, Labadie B, Shafran J, Rameshbabu S, Drovetsky C, Bynoe K, Solanki A, Markiewicz E, et al. Suppression of tumor cell lactate-generating signaling pathways eradicates murine PTEN/p53-deficient aggressive-variant prostate cancer via macrophage phagocytosis. Clin Cancer Res. 2023;29(23):4930–4940. 37721526 10.1158/1078-0432.CCR-23-1441 PMC10841690

[188] De Leo A, Ugolini A, Yu X, Scirocchi F, Scocozza D, Peixoto B, Pace A, D’Angelo L, Liu JKC, Etame AB, et al. Glucose-driven histone lactylation promotes the immunosuppressive activity of monocyte-derived macrophages in glioblastoma. Immunity. 2024;57(5):1105–1123.e8. 38703775 10.1016/j.immuni.2024.04.006 PMC11114377

[189] Li XM, Yang Y, Jiang FQ, Hu G, Wan S, Yan WY, He XS, Xiao F, Yang XM, Guo X, et al. Histone lactylation inhibits RARγ expression in macrophages to promote colorectal tumorigenesis through activation of TRAF6-IL-6-STAT3 signaling. Cell Rep. 2024;43(2): Article 113688. 38245869 10.1016/j.celrep.2024.113688

[190] Yang K, Fan M, Wang X, Xu J, Wang Y, Tu F, Gill PS, Ha T, Liu L, Williams DL, et al. Lactate promotes macrophage HMGB1 lactylation, acetylation, and exosomal release in polymicrobial sepsis. Cell Death Differ. 2022;29(1):133–146. 34363018 10.1038/s41418-021-00841-9 PMC8738735

[191] Pan RY, He L, Zhang J, Liu X, Liao Y, Gao J, Liao Y, Yan Y, Li Q, Zhou X, et al. Positive feedback regulation of microglial glucose metabolism by histone H4 lysine 12 lactylation in Alzheimer’s disease. Cell Metab. 2022;34(4):634–648.e6. 35303422 10.1016/j.cmet.2022.02.013

[192] Wei L, Yang X, Wang J, Wang Z, Wang Q, Ding Y, Yu A. H3K18 lactylation of senescent microglia potentiates brain aging and Alzheimer’s disease through the NFκB signaling pathway. J Neuroinflammation. 2023;20(1):208. 37697347 10.1186/s12974-023-02879-7 PMC10494370

[193] Chen AN, Luo Y, Yang YH, Fu JT, Geng XM, Shi JP, Yang J. Lactylation, a novel metabolic reprogramming code: Current status and prospects. Front Immunol. 2021;10(12): Article 688910. 10.3389/fimmu.2021.688910 PMC8222712 34177945

[194] Xie Y, Hu H, Liu M, Zhou T, Cheng X, Huang W, Cao L. The role and mechanism of histone lactylation in health and diseases. Front Genet. 2022;23(13): Article 949252. 10.3389/fgene.2022.949252 PMC9445422 36081996

[195] Zhang D, Gao J, Zhu Z, Mao Q, Xu Z, Singh PK, Rimayi CC, Moreno-Yruela C, Xu S, Li G, et al. Lysine L-lactylation is the dominant lactylation isomer induced by glycolysis. Nat Chem Biol. 2024;21(1):91–99. 39030363 10.1038/s41589-024-01680-8 PMC11666458

[196] Hingorani AD, Chan NN. D-lactate encephalopathy. Lancet. 2001;358(9295):1814. 10.1016/S0140-6736(01)06818-0 11734269

[197] McDonald B, Zucoloto AZ, Yu IL, Burkhard R, Brown K, Geuking MB, McCoy KD. Programing of an intravascular immune firewall by the gut microbiota protects against pathogen dissemination during infection. Cell Host Microbe. 2020;28(5):660–668.e4. 32810440 10.1016/j.chom.2020.07.014

[198] Locati M, Curtale G, Mantovani A. Diversity, mechanisms, and significance of macrophage plasticity. Annu Rev Pathol. 2020;24(15):123–147. 10.1146/annurev-pathmechdis-012418-012718 PMC7176483 31530089

[199] Scott EN, Gocher AM, Workman CJ, Vignali DAA. Regulatory T cells: Barriers of immune infiltration into the tumor microenvironment. Front Immunol. 2021;10(12): Article 702726. 10.3389/fimmu.2021.702726 PMC8222776 34177968

[200] Watson MJ, Vignali PDA, Mullett SJ, Overacre-Delgoffe AE, Peralta RM, Grebinoski S, Menk AV, Rittenhouse NL, DePeaux K, Whetstone RD, et al. Metabolic support of tumour-infiltrating regulatory T cells by lactic acid. Nature. 2021;591(7851):645–651. 33589820 10.1038/s41586-020-03045-2 PMC7990682

[201] Liu C, Chikina M, Deshpande R, Menk AV, Wang T, Tabib T, Brunazzi EA, Vignali KM, Sun M, Stolz DB, et al. Treg cells promote the SREBP1-dependent metabolic fitness of tumor-promoting macrophages via repression of CD8+ T cell-derived interferon-γ. Immunity. 2019;51(2):381–397.e6. 31350177 10.1016/j.immuni.2019.06.017 PMC6703933

[202] Gu J, Zhou J, Chen Q, Xu X, Gao J, Li X, Shao Q, Zhou B, Zhou H, Wei S, et al. Tumor metabolite lactate promotes tumorigenesis by modulating MOESIN lactylation and enhancing TGF-β signaling in regulatory T cells. Cell Rep. 2022;39(12): Article 110986. 35732125 10.1016/j.celrep.2022.110986

[203] Sun T, Liu B, Li Y, Wu J, Cao Y, Yang S, Tan H, Cai L, Zhang S, Qi X, et al. Oxamate enhances the efficacy of CAR-T therapy against glioblastoma via suppressing ectonucleotidases and CCR8 lactylation. J Exp Clin Cancer Res. 2023;42(1):253. 37770937 10.1186/s13046-023-02815-w PMC10540361

[204] Varner EL, Trefely S, Bartee D, von Krusenstiern E, Izzo L, Bekeova C, O’Connor RS, Seifert EL, Wellen KE, Meier JL, et al. Quantification of lactoyl-CoA (lactyl-CoA) by liquid chromatography mass spectrometry in mammalian cells and tissues. Open Biol. 2020;10(9): Article 200187. 32961073 10.1098/rsob.200187 PMC7536085

[205] Jin J, Bai L, Wang D, Ding W, Cao Z, Yan P, Li Y, Xi L, Wang Y, Zheng X, et al. SIRT3-dependent delactylation of cyclin E2 prevents hepatocellular carcinoma growth. EMBO Rep. 2023;24(5): Article e56052. 36896611 10.15252/embr.202256052 PMC10157311

[206] Wang J, Yang P, Yu T, Gao M, Liu D, Zhang J, Lu C, Chen X, Zhang X, Liu Y. Lactylation of PKM2 suppresses inflammatory metabolic adaptation in pro-inflammatory macrophages. Int J Biol Sci. 2022;18(16):6210–6225. 36439872 10.7150/ijbs.75434 PMC9682528

[207] Lopez Krol A, Nehring HP, Krause FF, Wempe A, Raifer H, Nist A, Stiewe T, Bertrams W, Schmeck B, Luu M, et al. Lactate induces metabolic and epigenetic reprogramming of pro-inflammatory Th17 cells. EMBO Rep. 2022;23(12): Article e54685. 36215678 10.15252/embr.202254685 PMC9724659

[208] Wang N, Wang W, Wang X, Mang G, Chen J, Yan X, Tong Z, Yang Q, Wang M, Chen L, et al. Histone lactylation boosts reparative gene activation post-myocardial infarction. Circ Res. 2022;131(11):893–908. 36268709 10.1161/CIRCRESAHA.122.320488

[209] Fan M, Yang K, Wang X, Chen L, Gill PS, Ha T, Liu L, Lewis NH, Williams DL, Li C. Lactate promotes endothelial-to-mesenchymal transition via Snail1 lactylation after myocardial infarction. Sci Adv. 2023;9(5): Article eadc9465. 36735787 10.1126/sciadv.adc9465 PMC9897666

[210] Zhu D, Liang H, Du Z, Liu Q, Li G, Zhang W, Wu D, Zhou X, Song Y, Yang C. Altered metabolism and inflammation driven by post-translational modifications in intervertebral disc degeneration. Research. 2024;5(7):0350. 10.34133/research.0350 PMC10997488 38585329

[211] Zhang W, Xu L, Yu Z, Zhang M, Liu J, Zhou J. Inhibition of the glycolysis prevents the cerebral infarction progression through decreasing the lactylation levels of LCP1. Mol Biotechnol. 2023;65(8):1336–1345. 36574182 10.1007/s12033-022-00643-5 PMC10352161

[212] Yao Y, Bade R, Li G, Zhang A, Zhao H, Fan L, Zhu R, Yuan J. Global-scale profiling of differential expressed lysine-lactylated proteins in the cerebral endothelium of cerebral ischemia-reperfusion injury rats. Cell Mol Neurobiol. 2023;43(5):1989–2004. 36030297 10.1007/s10571-022-01277-6 PMC11412193

[213] Sommer N, Alebrahimdehkordi N, Pak O, Knoepp F, Strielkov I, Scheibe S, Dufour E, Andjelković A, Sydykov A, Saraji A, et al. Bypassing mitochondrial complex III using alternative oxidase inhibits acute pulmonary oxygen sensing. Sci Adv. 2020;6(16): Article eaba0694. 32426457 10.1126/sciadv.aba0694 PMC7159913

[214] Southgate L, Machado RD, Gräf S, Morrell NW. Molecular genetic framework underlying pulmonary arterial hypertension. Nat Rev Cardiol. 2020;17(2):85–95. 31406341 10.1038/s41569-019-0242-x

[215] Hagihara H, Shoji H, Otabi H, Toyoda A, Katoh K, Namihira M, Miyakawa T. Protein lactylation induced by neural excitation. Cell Rep. 2021;37(2): Article 109820. 34644564 10.1016/j.celrep.2021.109820

[216] Kan RL, Chen J, Sallam T. Crosstalk between epitranscriptomic and epigenetic mechanisms in gene regulation. Trends Genet. 2022;38(2):182–193. 34294427 10.1016/j.tig.2021.06.014 PMC9093201

[217] Kolosenko I, Avnet S, Baldini N, Viklund J, De Milito A. Therapeutic implications of tumor interstitial acidification. Semin Cancer Biol. 2017;43:119–133. 28188829 10.1016/j.semcancer.2017.01.008

[218] Sun H, Zhu A, Zhou X, Wang F. Suppression of pyruvate dehydrogenase kinase-2 re-sensitizes paclitaxel-resistant human lung cancer cells to paclitaxel. Oncotarget. 2017;8(32):52642–52650. 28881758 10.18632/oncotarget.16991 PMC5581057

[219] Ding B, Zheng P, Tan J, Chen H, Meng Q, Li J, Li X, Han D, Li Z, Ma X, et al. Sodium bicarbonate nanoparticles for amplified cancer immunotherapy by inducing pyroptosis and regulating lactic acid metabolism. Angew Chem Int Ed Engl. 2023;62(40): Article e202307706. 37587061 10.1002/anie.202307706

[220] Jin H, Luo R, Li J, Zhao H, Ouyang S, Yao Y, Chen D, Ling Z, Zhu W, Chen M, et al. Inhaled platelet vesicle-decoyed biomimetic nanoparticles attenuate inflammatory lung injury. Front Pharmacol. 2022;13:1050224. 36523494 10.3389/fphar.2022.1050224 PMC9745055

[221] Feng F, Wu J, Chi Q, Wang S, Liu W, Yang L, Song G, Pan L, Xu K, Wang C. Lactylome analysis unveils lactylation-dependent mechanisms of stemness remodeling in the liver cancer stem cells. Adv Sci. 2024;5: Article e2405975. 10.1002/advs.202405975 PMC11481176 39099416

[222] Wang J, Liu Z, Xu Y, Wang Y, Wang F, Zhang Q, Ni C, Zhen Y, Xu R, Liu Q, et al. Enterobacterial LPS-inducible LINC00152 is regulated by histone lactylation and promotes cancer cells invasion and migration. Front Cell Infect Microbiol. 2022;25(12): Article 913815. 10.3389/fcimb.2022.913815 PMC9359126 35959377

[223] Chen B, Deng Y, Hong Y, Fan L, Zhai X, Hu H, Yin S, Chen Q, Xie X, Ren X, et al. Metabolic recoding of NSUN2-mediated m5C modification promotes the progression of colorectal cancer via the NSUN2/YBX1/m5C-ENO1 positive feedback loop. Adv Sci. 2024;11(28): Article e2309840. 10.1002/advs.202309840 PMC11267267 38769664

[224] Chen Y, Wu J, Zhai L, Zhang T, Yin H, Gao H, Zhao F, Wang Z, Yang X, Jin M, et al. Metabolic regulation of homologous recombination repair by MRE11 lactylation. Cell. 2024;187(2):294–311.e21. 38128537 10.1016/j.cell.2023.11.022 PMC11725302

[225] Chen J, Zhao D, Wang Y, Liu M, Zhang Y, Feng T, Xiao C, Song H, Miao R, Xu L, et al. Lactylated apolipoprotein C-II induces immunotherapy resistance by promoting extracellular lipolysis. Adv Sci. 2024;9: Article e2406333. 10.1002/advs.202406333 PMC11481198 38981044

[226] Yan F, Teng Y, Li X, Zhong Y, Li C, Yan F, He X. Hypoxia promotes non-small cell lung cancer cell stemness, migration, and invasion via promoting glycolysis by lactylation of SOX9. Cancer Biol Ther. 2024;25(1):2304161. 38226837 10.1080/15384047.2024.2304161 PMC10793688

[227] Gu X, Zhuang A, Yu J, Yang L, Ge S, Ruan J, Jia R, Fan X, Chai P. Histone lactylation-boosted ALKBH3 potentiates tumor progression and diminished promyelocytic leukemia protein nuclear condensates by m1A demethylation of SP100A. Nucleic Acids Res. 2024;52(5):2273–2289. 38118002 10.1093/nar/gkad1193 PMC10954454

[228] Liu R, Zou Z, Chen L, Feng Y, Ye J, Deng Y, Zhu X, Zhang Y, Lin J, Cai S, et al. FKBP10 promotes clear cell renal cell carcinoma progression and regulates sensitivity to the HIF2α blockade by facilitating LDHA phosphorylation. Cell Death Dis. 2024;15(1):64. 38233415 10.1038/s41419-024-06450-x PMC10794466

[229] Fan W, Zeng S, Wang X, Wang G, Liao D, Li R, He S, Li W, Huang J, Li X, et al. A feedback loop driven by H3K9 lactylation and HDAC2 in endothelial cells regulates VEGF-induced angiogenesis. Genome Biol. 2024;25(1):165. 38918851 10.1186/s13059-024-03308-5 PMC11197246

[230] Meng Y, Fan XY, Yang LJ, Xu BQ, He D, Xu Z, Wu D, Wang B, Cui HY, Wang SJ, et al. Detachment activated CyPA/CD147 induces cancer stem cell potential in non-stem breast cancer cells. Front Cell Dev Biol. 2020;16(8): Article 543856. 10.3389/fcell.2020.543856 PMC7640948 33195186

[231] Thongon N, Zucal C, D’Agostino VG, Tebaldi T, Ravera S, Zamporlini F, Piacente F, Moschoi R, Raffaelli N, Quattrone A, et al. Cancer cell metabolic plasticity allows resistance to NAMPT inhibition but invariably induces dependence on LDHA. Cancer Metab. 2018;(6):1. 29541451 10.1186/s40170-018-0174-7 PMC5844108

